# The inverse association between skeletal muscle mass to visceral fat ratio (SVR) and sleep disturbance: the mediating role of inflammation and aging acceleration

**DOI:** 10.1186/s12888-026-08248-x

**Published:** 2026-06-05

**Authors:** Li Zhang, QianKun Yang, XiXi Wu, Yue Hu, Jie Yu

**Affiliations:** 1 Department of Hematology and Oncology of Children’s Hospital of Chongqing Medical University‚ National Clinical Research Center for Children and Adolescents‘ Health and Diseases‚ Ministry of Education Key Laboratory of Child Development and Disorders, Chongqing Key Laboratory of Pediatric Metabolism and Inflammatory Diseases, No.136 of Zhongshan Second road‚ YuZhong District, Chongqing, 400014 China; 2 Department of Orthopedics, Southwest Hospital, Army Medical University, Chongqing, 400038 China; 3 Ross University School of Medicine, St Michael, Barbados; 4 Department of Neurology, Children’s Hospital of Chongqing Medical University, National Clinical Research Center for Child Health and Disorders, Ministry of Education Key Laboratory of Child Development and Disorders, Chongqing Key Laboratory of Pediatrics, No.136 of Zhong shan Second Road, Yu zhong District, Chongqing, 400014 China

**Keywords:** Sarcopenic obesity, SVR, Sleep disturbance, Inflammation, PhenoAgeAccel

## Abstract

**Background:**

A growing body of evidence has linked body composition to poor sleep quality. The skeletal muscle mass–to–visceral fat ratio (SVR) is a valid indicator of body composition that integrates appendicular skeletal muscle mass and visceral fat, reflecting the severity of sarcopenic obesity (SO). However, the association between SVR and sleep disturbance has not been well established. This study aimed to investigate the relationship between SVR and sleep disturbance among U.S. adults, along with the mediating roles of inflammation and biological aging.

**Methods:**

A total of 1,564 participants from the National Health and Nutrition Examination Survey (NHANES) with complete data on SVR, sleep parameters, and other essential covariates were included. Weighted multivariable logistic regression models, subgroup and interaction analyses, and restricted cubic spline (RCS) analyses were used to explore the association between SVR and sleep disturbance. Furthermore, mediation analysis was used to explore the mediating role of aggregate index of systemic inflammation (AISI), C-reactive Protein-Albumin-Lymphocyte index (CALLY), and Phenotypic age acceleration (PhenoAgeAccel) in this association.

**Results:**

15.22% of participants reported sleep disturbance. After full covariate adjustment, SVR was inversely associated with sleep disturbance: each unit increase in SVR was linked to a 91.1% lower risk (OR = 0.089, 95% CI: 0.024–0.334), and the highest versus lowest SVR quartile showed a 57.4% reduced risk (OR = 0.426, 95% CI: 0.241–0.752; P-trend = 0.006). RCS analyses confirmed a significant linear dose-response relationship between SVR and sleep disturbance (P-nonlinearity = 0.907). Subgroup analyses and interaction tests showed a more pronounced association in females (OR = 0.42 vs. males: OR = 0.01) and individuals with depression (OR = 0.16 vs. non-depressed individuals: OR < 0.01). Additionally, after adjusting for alternative mediators in sensitivity analyses, AISI, CALLY, and PhenoAgeAccel statistically mediated 10.64%, 36.43%, and 15.99% of the association, respectively (all *P* < 0.05).

**Conclusions:**

SVR was inversely associated with sleep disturbance, with inflammation and biological aging statistically explaining a significant portion of this association. Further studies with longitudinal or interventional designs are warranted to clarify the causal links of body composition with inflammation, biological aging and sleep disturbance given the cross-sectional design.

**Clinical trial number:**

Not applicable.

**Supplementary Information:**

The online version contains supplementary material available at 10.1186/s12888-026-08248-x.

## Introduction

Sleep plays a pivotal role in physical and mental well-being. However, sleep disturbance, characterized by disrupted sleep patterns or reduced sleep quality, has become an increasingly important global public health concern. It is estimated that 30%~50% of the global population experiences sleep disturbance, incurring annual health burden costs of approximately $3,400–$5,200 per person [1–3]. Moreover, sleep disturbance has been associated with a wide range of adverse health outcomes, including diabetes [4], depression [5], and hypertension [6]. Therefore, identifying risk factors and clarifying the underlying mechanisms of sleep disturbance are of considerable importance for improving sleep health and reducing its related disease burden.

Sleep disturbance is influenced by multiple factors, including biological alterations in sleep propensity, psychosocial stress, and substance use [7–10]. Recently, a growing body of evidence has revealed a close association between body composition and sleep disturbance, particularly involving sarcopenia and adiposity accumulation. Notably, the relationship between sarcopenia and sleep disturbance appears to be bidirectional. On the one hand, reduced muscle mass and strength are consistently associated with poor sleep quality. Sarcopenic individuals have been shown to associate with increased odds ratio (OR) of sleep disorders (OR = 1.732, 95%CI: 1.182–2.547) [11], with particularly pronounced associations observed in older diabetic populations (OR = 6.04, 95%CI: 1.71–21.36 for men, OR = 6.33, 95%CI: 1.91–20.97 for women) [12]. A systematic review of 24 studies further confirmed a significant positive association between weak muscle strength and poor sleep quality or duration in older adults [13]. Sarcopenia has also been linked to various neurological conditions that may secondarily affect sleep, including depression, Parkinson’s disease, and cognitive impairment [14]. On the other hand, poor sleep appears to exacerbate muscle loss. Both short and long sleep durations have been associated with a higher prevalence of sarcopenia [15, 16], and lower muscle quality indices associate with increased sleep complaints in large population-based cohorts [17]. Longitudinal evidence from the CHARLS study further indicates that short sleep duration predicts an increased risk of developing sarcopenia and declining handgrip strength over time [18, 19].

Similarly, a bidirectional relationship has been documented between adiposity accumulation and sleep disturbance. On the one hand, poor sleep parameters appear to promote visceral fat accumulation. Experimental sleep restriction (4 hours’ sleep) has been shown to increase caloric intake and abdominal adiposity compared with normal sleep (9 hours’ sleep) [20], and longitudinal data confirm that both short (≤ 6 h/day) and long (≥ 9 h/day) sleep durations are associated with greater visceral adipose tissue accumulation over time relative to normal sleepers (7–8 h per night) [21]. On the other hand, excess adiposity, particularly visceral fat, is associated with an increased susceptibility to sleep disturbance. Multiple obesity-related indices, including the body roundness index (BRI) [22], metabolic score for visceral fat (METS-VF) [23], and visceral adiposity index (VAI) [24], have been linked to a higher prevalence of sleep complaints. Similar associations have been observed between obstructive sleep apnea and fat accumulation markers, such as VAI, atherogenic index of plasma (AIP), and lipid accumulation product (LAP) [25–27].

Sarcopenic obesity (SO) is a metabolic condition characterized by the coexistence of skeletal muscle loss and excessive adipose tissue accumulation, particularly visceral fat. Compared with either sarcopenia or obesity alone, SO may confer a greater risk of cardiometabolic dysfunction and adverse health outcomes [28–31]. The interplay between muscle loss and fat accumulation creates a self-reinforcing pathological vicious cycle: excessive adipose tissue initiates chronic, low-grade inflammation, inducing muscle atrophy and compromising muscle regenerative capacity [32, 33]. Accordingly, reduced muscle mass often promotes physical inactivity and induces negative energy expenditure balance, ultimately leading to further exacerbating fat accumulation [34]. The skeletal muscle mass to visceral fat area ratio (SVR) is a composite indicator reflecting the balance between appendicular skeletal muscle mass (ASM) and visceral fat area (VFA), and has been increasingly used to evaluate the risk of body composition imbalance in conditions such as SO. SVR has been shown to be associated with various diseases, including metabolic dysfunction-associated fatty liver disease [35, 36], depression [32], diabetes and metabolic syndrome [37], and cognitive impairment [38]. Many of these conditions are also known to be directly or indirectly associated with poor sleep quality or sleep disorders. Nevertheless, whether a specific relationship exists between SVR and sleep disorder remains unclear.

Accumulating evidence suggests that SO and sleep disturbance may share common biological pathways rather than representing independent conditions. SO is increasingly recognized as an aging-related syndrome characterized by chronic low-grade inflammation (“inflammaging”), hormonal alterations, adipokine imbalance (elevated leptin/reduced adiponectin), and insulin resistance [39, 40]. Specifically, excessive visceral adipose tissue can secrete pro-inflammatory cytokines (e.g., IL-6, TNF-α) and lipotoxic free fatty acids, which leads to the exacerbation of inflammation and biological aging [41, 42]. In parallel, sleep disturbance (e.g., insufficient sleep, sleep fragmentation, obstructive sleep apnea) have been consistently shown to be key initiators of the vicious cycle of systemic inflammation and accelerated aging, via mechanisms involving inflammatory activation linked to circadian disruption and immune dysregulation [43, 44]. Collectively, these observations suggest that systemic inflammation and biological aging may constitute shared mechanistic pathways linking alterations in body composition to impaired sleep physiology. Therefore, indicators reflecting systemic inflammatory burden (e.g., aggregate index of systemic inflammation [AISI], C-reactive protein–albumin–lymphocyte index [CALLY]) and biological aging (e.g., phenotypic age acceleration [PhenoAgeAccel]) may serve as potential mediators in the association between SVR and sleep disturbance, representing a hypothesis that merits empirical investigation.

Given this, the present study aimed to investigate the association between SVR and sleep disorders using data from the NHANES database. As a secondary aim, mediation analysis was conducted to examine the potential mediating effects of AISI, CALLY, and PhenoAgeAccel in the association between SVR and sleep disorders. Our findings may contribute to a better understanding of the link between body composition and sleep disorders and provide insights for the development of preventive and therapeutic strategies to improve sleep quality.

## Methods

### Study design and study population

Our study was conducted based on the publicly available National Health and Nutrition Examination Survey (NHANES) database, which aimed to assess the health and nutritional status of people in communities in the United States [45]. NHANES employs a method of stratified multistage probability sampling to ensure its national representativeness. All NHANES survey protocols are reviewed and approved by the National Center for Health Statistics (NCHS) Ethics Review Board (ERB), and all participants have signed the informed consent before their participation. Moreover, since NHANES database is publicly available, the additional ethical or administrative permissions are thus waived.

**Fig. 1:**
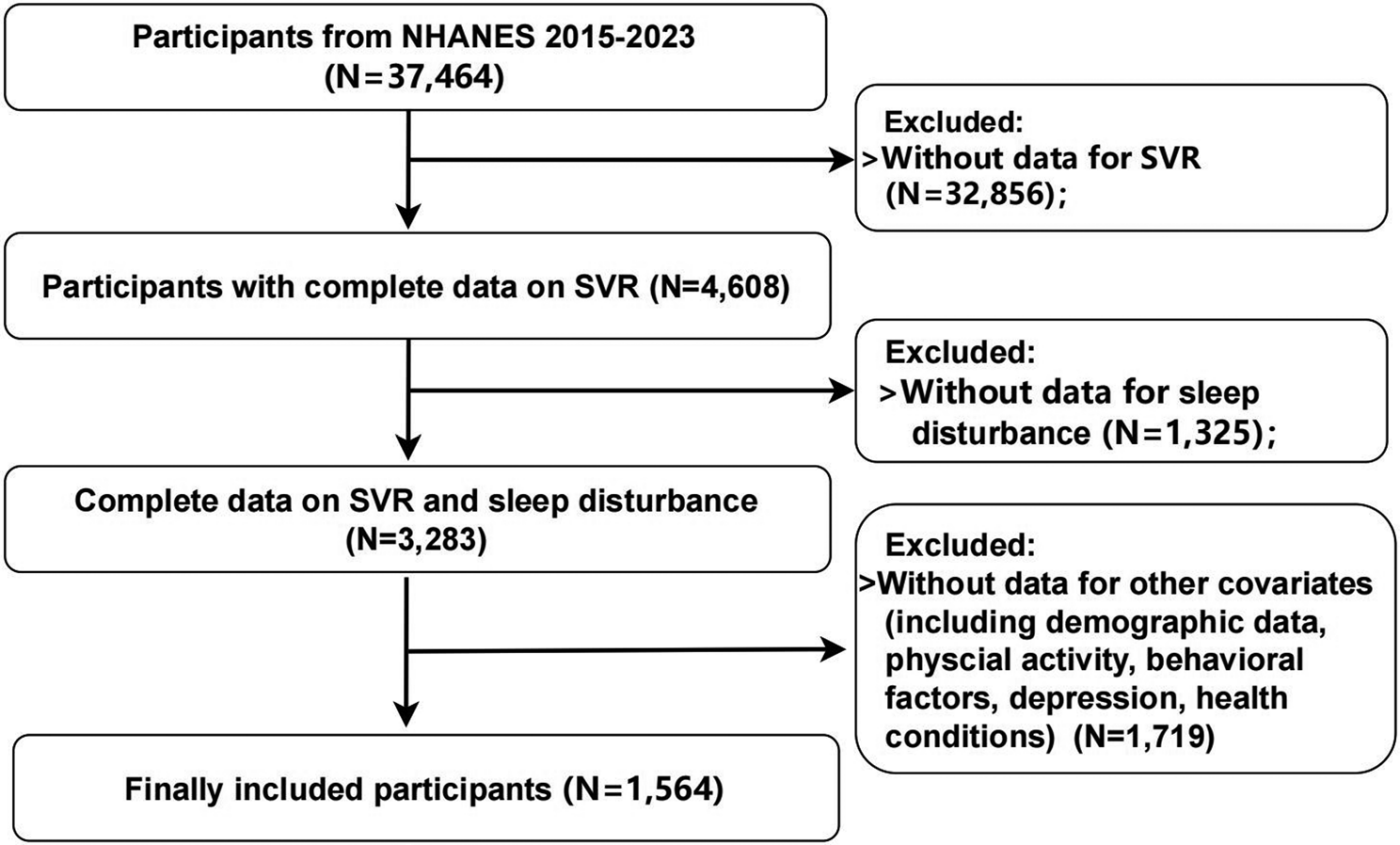
Flow chart of participants selection

Participants from the NHANES database between 2015 and 2023 were included in this study. After excluding those without complete information on skeletal muscle mass to visceral fat area ratio (SVR) (*n* = 32,856), sleep parameters (*n* = 1,325), and essential covariates (*n* = 1,719), a total of 1,564 participants were finally enrolled in this study. The flow chart of participant selection was presented in Fig. 1.

### Definition of sleep disturbance

Sleep disturbance was defined using data from the “Sleep Disorders” module of the NHANES as previously described [46]. The Sleep Disorders questionnaire consists of ten items covering usual sleep time/wake time/sleep hours on weekdays/workdays and weekends, and existence of snoring, snorting or stop breathing, trouble sleeping, and feeling of overly sleepy during day [47]. Five components were used to define sleep disturbance (STable 1): (1) weekdays/workdays sleep duration (categorized as short <7 h, normal 7–9 h, long>9 h; assessed via SLD012 - Sleep hours - weekdays or workdays), (2) trouble sleeping (assessed via SLQ050: “Ever told a doctor about trouble sleeping?“), (3) snoring frequency (SLQ030: “How often do you snore?“), (4) excessive daytime sleepiness (SLQ120: “How often feel overly sleepy during the day?“), and (5) sleep apnea-related symptoms (SLQ040: “How often do you snort or stop breathing?“). Each component was scored on a 0–1 scale as detailed in Supplementary Table 1, with total scores ranging from 0 to 5 (lower scores indicating worse sleep quality). Individuals with a total score < 2 were classified as having sleep disturbance [46].

### Assessment of SVR

The modules of “Dual-Energy X-ray Absorptiometry - Whole Body” and “Dual-Energy X-ray Absorptiometry - Android/Gynoid Measurements” in NHANES are used to get the data of appendicular skeletal muscle mass (ASM) and visceral fat area (VFA), respectively. ASM is calculated as the sum of lean mass (excluding bone mineral content) from the four extremities (upper and lower limbs, measured in kg). Visceral adipose tissue area is measured at the intervertebral space between the fourth and fifth lumbar vertebrae. Skeletal muscle mass to visceral fat area ratio (SVR) can be calculated using the following formula [32]: SVR = ASM/VFA (kg/cm^2^).

### Assessment of AISI, CALLY and PhenoAgeAccel

Aggregate index of systemic inflammation (AISI) was calculated as previously described [48]. Briefly, AISI was derived by the product of platelet, neutrophil, and monocyte counts by the lymphocyte count. The calculation of C-reactive Protein-Albumin-Lymphocyte (CALLY) index was described detailedly in previous studies [49]. The CALLY index is defined as the ratio of albumin (g/L) multiplied by lymphocyte count (1000 cells/µL) to CRP (mg/dL). Ten variables, including chronological age, albumin, creatinine, glucose, C-reactive protein, lymphocyte percentage, mean cell volume, red blood cell distribution width, alkaline phosphatase, and white blood cell count, were utilized to calculate Phenotypic age (PhenoAge) as described by Morgan E. Levine et al. [50]. PhenoAge Acceleration (PhenoAgeAccel) represents the residual derived from regressing Phenotypic Age onto chronological age in a linear model [51]. A positive PhenoAgeAccel value indicates an individual’s physiological status exceeds what he/she should be relative to their actual years, suggesting an accelerated status of biological aging.

### Covariates

Covariates were selected based on evidence from previous studies and clinical considerations [32, 46]. Age, gender, race, education, marital status, ratio of family income to poverty (PIR), smoking, drinking, physical activity (PA), cardiovascular disease (CVD), cancer, hypertension, diabetes, depression, and hyperlipemia were included as essential covariates in this study. Specifically, age and PIR were treated as continuous variables and subsequently divided into two (< 40, ≥ 40 years) and three categories (< 1.3, 1.3–3.5, ≥ 3.5) as previously described [46, 52], respectively. Gender included two classifications, namely male and female, while race was categorized into five groups, including non-Hispanic white, non-Hispanic black, Mexican American, other Hispanic, and other race. Marital status was classified into married/living with partner versus living alone, following established criteria [53]. Education level was classified into the following groups as previously described [54]: high school, high school or equivalent, and college or above. Smoking status was divided into three groups based on their self-reported smoking status, including never smokers, former smokers, and current smokers. Individuals who claimed to have less than 100 cigarettes in their lives were defined as never smokers, and individuals who had reached the criteria of smoking 100 cigarettes in their lives but had quit smoking now were labelled as former smokers, while who were currently smoking were defined as current smokers [55]. For alcohol consumption status, those who had taken in ≤ 1 drink/day in women and ≤ 2 drinks/day in men were defined as mild drinking, and those who had taken in 1–3 drinks/day in women and 2–4 drinks/day in men were classified as moderate drinking, while those who drunk as ≥ 4 drinks/day in women and ≥ 5 drinks/day in men were categorized into heavy drinking [56]. The leisure-time physical activity (PA) was assessed based on the information from Global Physical Activity Questionnaire (GPAQ), which had taken into account the frequency (sessions per week) and duration (minutes per session) of moderate and vigorous PA. Previous studies have revealed that one minute of vigorous PA is equivalent to two minutes’ moderate PA [52, 57, 58]. Therefore, the weekly PA was calculated as vigorous PA (minutes) + moderate PA (minutes) [52, 57, 58]. Participants were classified into low (< 500 MET-minutes per week) and high PA (≥ 500 MET-minutes per week) groups based on the threshold of 500 MET-minutes/week from the national physical activity guidelines [59, 60]. Five major clinical events were used to identify cardiovascular disease (CVD) as previously reported [54, 57], including congestive heart failure (CHF), coronary heart disease (CHD), angina, cardiac arrest, and stroke. Individuals who answered “yes” to the question of “Have you ever been told by a physician that you had CHD/CHF/angina/a heart attack or a stroke?” were deemed as having CVD [54]. Individuals who had reached one or more of the following criteria were diagnosed with hypertension [61], including self-reported physician-diagnosed hypertension, average systolic/diastolic blood pressure ≥ 130/80 mmHg, and use of antihypertensive currently. Similarly, individuals who had met any of the following criteria were defined as having diabetes [62], including HbA1C ≥ 6.5%/FPG ≥ 7.00 mmol/L, self-reported clinician-diagnosed diabetes, use of insulin/antidiabetic medication currently, OGTT ≥ 11.10 mmol/L, and random blood glucose ≥ 11.10 mmol/L. Cancer can be identified if individuals answered “yes” to the question of “MCQ220 - Ever told you had cancer or malignancy?”. The 9-item Patient Health Questionnaire (PHQ-9) was utilized to assessed depression, which was identified by using a standard cut point of PHQ-9 scores ≥ 10 [63]. The diagnosis of hyperlipemia can be reached if individuals met any of the following criteria [64], including TC ≥ 200 mg/dL, TG ≥ 150 mg/dL, LDL-C ≥ 130 mg/dL, HDL-C ≤ 40 mg/dL (males) or ≤ 50 mg/dL (females), and taking lipid-lowering medications currently.

### Statistical analysis

All statistical analyses accounted for adjusted weights due to the complex multistage sampling design of NHANES. Variables including age, PIR, VFA, ASM, and SVR were presented as median (interquartile range, IQR) because of their skewed distributions. Differences between groups with and without sleep disturbance were compared using the Mann-Whitney U test. Categorical variables were reported as numbers (percentages), and intergroup differences were assessed via chi-square tests.

To explore the association between SVR and sleep disturbance, three weighted multivariate logistic regression models were established: Model 1, adjusted for no covariate; Model 2, adjusted for variables of age, gender, race, education, marital status, and PIR; Model 3, adjusted for variables of age, gender, race, education, marital status, PIR, smoking, drinking, PA, CVD, cancer, hypertension, diabetes, depression, and hyperlipemia. Additionally, adjusted restricted cubic spline (RCS) analysis—with adjustments matching those in Model 3—was employed to identify potential nonlinear relationship between SVR and sleep disturbance. Subgroup analyses and interaction tests were conducted to verify the robustness and consistency of the association. Stratified RCS analyses were performed if significant interactions were detected.

Mediation analyses were conducted to evaluate the potential mediating roles of systemic inflammation (AISI, CALLY) and biological aging (PhenoAgeAccel) in the association between SVR and sleep disturbance. First, Pearson correlation analysis was performed to assess correlations among the these mediators. Multicollinearity was evaluated using the ‘car’ R package, with a variance inflation factor (VIF) > 5 indicating potential collinearity [65]. We then performed mediation analyses using the ‘mediation’ R package, following established procedures [66]. In the primary analysis, separate single-mediator models were fitted for each candidate mediator (AISI, CALLY, and PhenoAgeAccel). For each model, a mediator model (SVR → mediator) and an outcome model (SVR + mediator → sleep disturbance) were specified, adjusting for the same covariates as in Model 3 (Table 2). The average causal mediation effect (ACME) and proportion mediated (defined as the ratio of the indirect effect to the total effect) were estimated for each mediator. Importantly, these single-mediator models estimate the total mediating effect of each variable separately and do not account for potential overlap or interrelationships among mediators. Therefore, the estimated indirect effects may not be independent, and the corresponding mediation proportions should not be interpreted as additive. To further assess robustness and explore the independent contribution of each mediator, sensitivity analyses were conducted by additionally adjusting for the remaining mediator(s) in the outcome model. These analyses provide conditional indirect effects, reflecting the mediating role of each variable after accounting for the others. The stability of mediation estimates was evaluated by comparing indirect effect sizes across models, with a relative change of less than 20% considered indicative of robustness. VIF values remained below 5 in all models, suggesting that multicollinearity did not materially affect the results.

All statistical procedures were conducted using R software (version 4.3.2), and statistical significance was defined as *P* < 0.05.

## Results

### Baseline characteristics

**Fig. 2:**
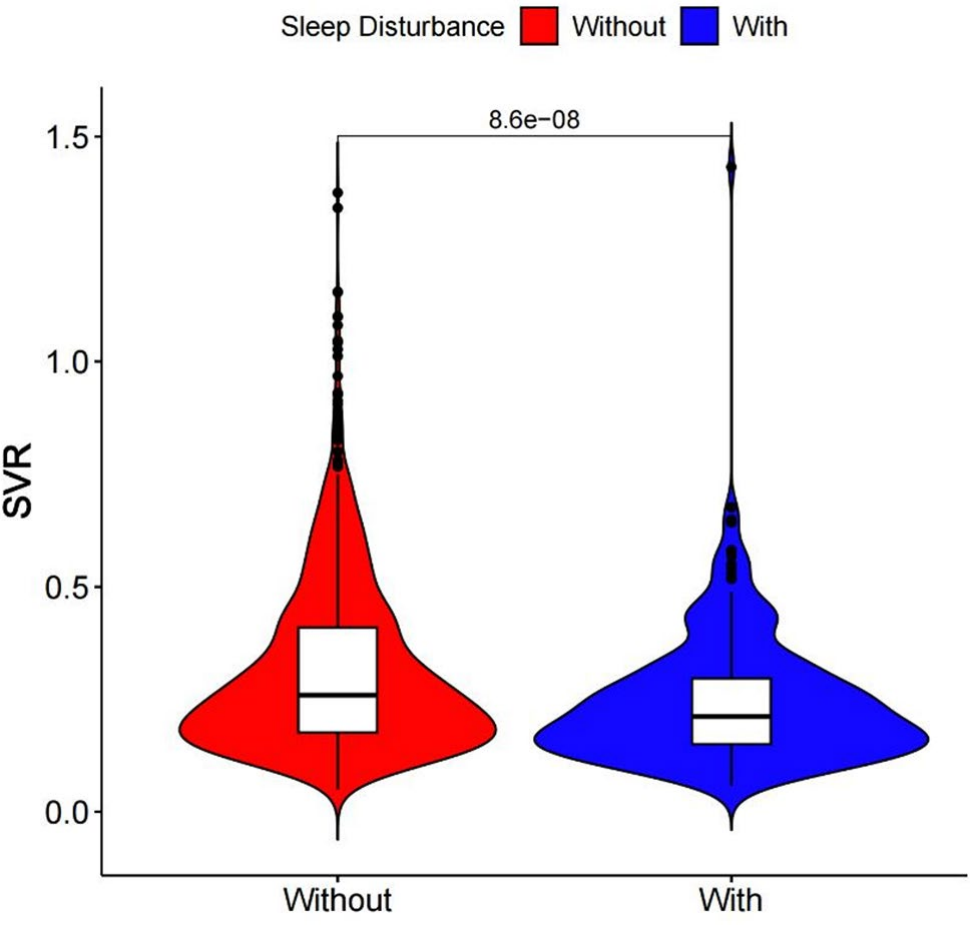
The violin plot reveals the difference of SVR between individuals with and without sleep disturbance

A total of 1,564 participants were finally included in this study, with 46.99% of them being female and 53.01% being male. The median (IQR) of age, VFA, ASM, and SVR for all participants were 37 (28–48) years, 93.35 (58.71-137.19) cm^2^, 23.41 (18.66–27.96) kg, and 0.25 (0.17–0.39) kg/cm^2^, respectively. The baseline characteristics of the study population were presented in Table 1. No significant differences were observed between groups in variables of gender, race, marital status, education, drinking status, and physical activity (all *P* > 0.05). Compared with those without sleep disturbance (84.78%), individuals with sleep disturbance (15.22%) were more likely to being current smokers, having morbidities of hypertension, diabetes, depression, hyperlipidemia, asthma, cancer, and CVD (all *P* < 0.05) (Table 1). Overall, SVR exhibited a skewed distribution, and only a small number of participants (12/1564) with SVR greater than 1 (SFig. 1). Notably, individuals with sleep disturbance exhibited a significantly lower level of SVR as opposed to those without sleep disturbance (*P* < 0.05) (Fig. 2). Additionally, individuals in the sleep disturbance group also showed a significantly lower sleep quality scores (0.824 ± 0.382 vs. 3.178 ± 0.911), and higher proportions of abnormal sleep duration (< 7 or > 9 h) (70.588% vs. 25.038%), trouble sleeping (71.008% vs. 15.535%), snoring (98.739% vs. 69.231%), excessive daytime sleep (97.479% vs. 55.279%), and sleep apnea symptoms (79.832% vs. 17.119% ) (all *P* < 0.05) (Table 2).

**Table 1:** Baseline characteristics of the study population

Characteristics	Overall (*n* = 1564)	Sleep disturbance		
		Without (*n* = 1326)	With (*n* = 238)	*P*-value
**Age**,** years**	37.00 (28.00–48.00)	37.00 (28.00–47.00)	41.00 (32.00–50.00)	< 0.001
**VFA**,** cm**^**2**^	93.35 (58.71-137.19)	89.49 (54.30-132.05)	118.70 (81.94-169.04)	< 0.001
**ASM**,** Kg**	23.41 (18.66–27.96)	23.17 (18.51–27.43)	25.67 (20.14–29.95)	< 0.001
**SVR**,** Kg/cm**^**2**^	0.25 (0.17–0.39)	0.26 (0.18–0.41)	0.21 (0.15–0.30)	< 0.001
**PIR**	2.44 (1.27–4.37)	2.47 (1.32–4.46)	2.12 (1.07–3.45)	0.002
**Gender**,** %**				0.334
Male	829 (53.01%)	696 (52.49%)	133 (55.88%)	
Female	735 (46.99%)	630 (47.51%)	105 (44.12%)	
**Race**,** %**				0.352
Mexican American	262 (16.75%)	229 (17.27%)	33 (13.87%)	
Other Hispanic	191 (12.21%)	156 (11.76%)	35 (14.71%)	
Non-Hispanic White	554 (35.42%)	467 (35.22%)	87 (36.55%)	
Non-Hispanic Black	315 (20.14%)	263 (19.83%)	52 (21.85%)	
Other Races	242 (15.47%)	211 (15.91%)	31 (13.03%)	
**Marital status**,** %**				0.49
Married or living with a partner	984 (62.92%)	839 (63.27%)	145 (60.92%)	
Living alone	580 (37.08%)	487 (36.73%)	93 (39.08%)	
**Education**,** %**				0.259
Less than high school	220 (14.07%)	185 (13.95%)	35 (14.71%)	
High school or equivalent	355 (22.70%)	292 (22.02%)	63 (26.47%)	
College or above	989 (63.24%)	849 (64.03%)	140 (58.82%)	
**Smoking**,** %**				< 0.001
Never	901 (57.61%)	798 (60.18%)	103 (43.28%)	
Former	270 (17.26%)	224 (16.89%)	46 (19.33%)	
Now	393 (25.13%)	304 (22.93%)	89 (37.39%)	
**Drinking**,** %**				0.821
Mild drinking	672 (42.97%)	572 (43.14%)	100 (42.02%)	
Moderate drinking	561 (35.87%)	477 (35.97%)	84 (35.29%)	
Heavy drinking	331 (21.16%)	277 (20.89%)	54 (22.69%)	
**PA categorical**,** %**				0.915
Low PA	425 (27.17%)	361 (27.22%)	64 (26.89%)	
High PA	1139 (72.83%)	965 (72.78%)	174 (73.11%)	
**Hypertension**,** %**				< 0.001
No	1156 (73.91%)	1028 (77.53%)	128 (53.78%)	
Yes	408 (26.09%)	298 (22.47%)	110 (46.22%)	
**Diabetes**,** %**				< 0.001
No	1395 (89.19%)	1199 (90.42%)	196 (82.35%)	
Yes	169 (10.81%)	127 (9.58%)	42 (17.65%)	
**Depression**,** %**				< 0.001
No	1447 (92.52%)	1252 (94.42%)	195 (81.93%)	
Yes	117 (7.48%)	74 (5.58%)	43 (18.07%)	
**Hyperlipidemia**,** %**				< 0.001
No	667 (42.65%)	593 (44.72%)	74 (31.09%)	
Yes	897 (57.35%)	733 (55.28%)	164 (68.91%)	
**Asthma**,** %**				0.038
No	1325 (84.72%)	1134 (85.52%)	191 (80.25%)	
Yes	239 (15.28%)	192 (14.48%)	47 (19.75%)	
**Cancer**,** %**				0.004
No	1509 (96.48%)	1287 (97.06%)	222 (93.28%)	
Yes	55 (3.52%)	39 (2.94%)	16 (6.72%)	
**CVD**,** %**				< 0.001
No	1520 (97.19%)	1297 (97.81%)	223 (93.70%)	
Yes	44 (2.81%)	29 (2.19%)	15 (6.30%)	

Note: Variables of age, PIR, VFA, ASM and SVR were presented as Median (Q1-Q3) due to their non-normal distribution characteristics. Abbreviations: VFA, Visceral adipose tissue area; ASM, appendicular skeletal muscle mass; SVR, skeletal muscle mass to visceral fat area ratio; PIR, ratio of family income to poverty; CVD, cardiovascular disease; PA, physical activity; MET, metabolic equivalent

**Table 2:** Sleep parameter based on the status of sleep disturbance

Characteristics	Overall	sleep disturbance		*P*-value
		Without	With	
**Sleep quality score**	2.820 (1.201)	3.178 (0.911)	0.824 (0.382)	< 0.001
**Sleep duration**				< 0.001
< 7 or > 9	500 (31.969%)	332 (25.038%)	168 (70.588%)	
7–9	1064 (68.031%)	994 (74.962%)	70 (29.412%)	
**Trouble sleeping**				< 0.001
No	1189 (76.023%)	1120 (84.465%)	69 (28.992%)	
Yes	375 (23.977%)	206 (15.535%)	169 (71.008%)	
**Snoring**				< 0.001
Never	411 (26.279%)	408 (30.769%)	3 (1.261%)	
Rarely/occasionally/frequently	1153 (73.721%)	918 (69.231%)	235 (98.739%)	
**Excessive daytime sleep**				< 0.001
Never/rarely	599 (38.299%)	593 (44.721%)	6 (2.521%)	
Rarely/sometimes/occasionally/frequently	965 (61.701%)	733 (55.279%)	232 (97.479%)	
**Sleep apnea symptoms**				< 0.001
Never	1147 (73.338%)	1099 (82.881%)	48 (20.168%)	
Rarely/sometimes/occasionally/frequently	417 (26.662%)	227 (17.119%)	190 (79.832%)	

Note: Sleep quality score were presented as Mean(SD), and other variables were presented as number (percentage)

### Association between SVR and sleep disturbance

**Fig. 3:**
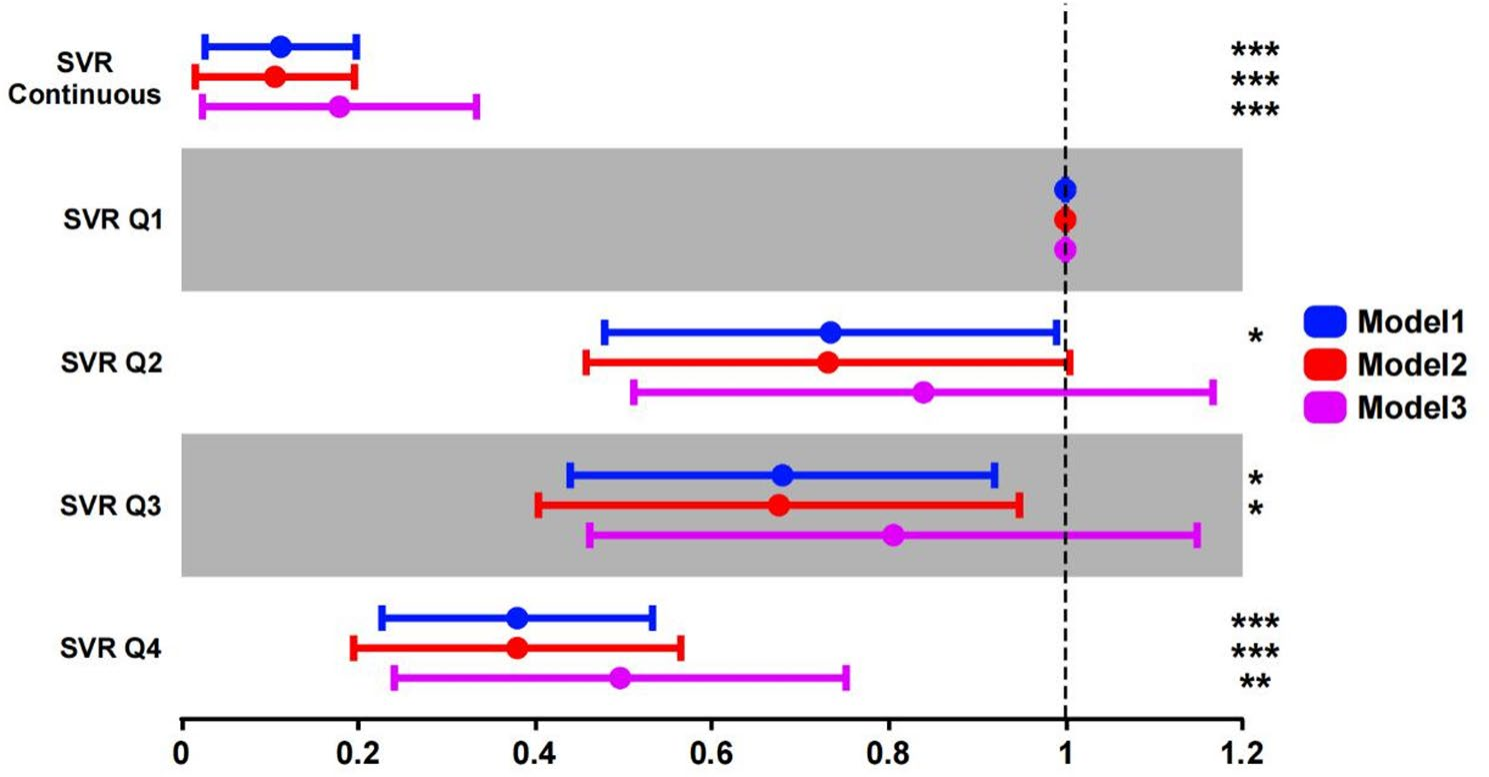
The association between SVR and sleep disturbance (related to Table 3). Abbreviations: SVR, skeletal muscle mass to visceral fat area ratio; Q, quartile

The multivariable logistic regression analyses were used to identify the relationship between SVR and sleep disturbance. As presented in Table 3; Fig. 3, whether SVR was analyzed as a continuous or a quartile variable, SVR was observed to significantly associate with reduced risks for sleep disturbance. In the fully-adjusted model, one-unit increase of SVR was associated with approximately 91.1% decreased risk for sleep disturbance (OR = 0.089, 95%CI : 0.024–0.334). When analyzed as a quartile variable, individuals in the highest quartile group (Q4) exhibited 57.4% reduced risk for sleep disturbance as opposed to individuals in the lowest quartile group (Q1) (P for trend = 0.00631).

**Table 3:** Association between SVR and sleep disturbance

Exposure	Model 1 OR (95%CI) Pvalue	Model 2 OR (95%CI) Pvalue	Model 3 OR (95%CI) Pvalue
**SVR**	0.073 (0.027, 0.198) < 0.00001	0.056 (0.016, 0.196) < 0.00001	0.089 (0.024, 0.334) 0.00034
**SVR quartile**			
Q1	1	1	1
Q2	0.689 (0.479, 0.990) 0.04416	0.678 (0.458, 1.005) 0.05309	0.773 (0.512, 1.167) 0.21998
Q3	0.637 (0.440, 0.920) 0.01638	0.619 (0.404, 0.948) 0.02742	0.728 (0.462, 1.149) 0.17266
Q4	0.348 (0.227, 0.533) < 0.00001	0.332 (0.195, 0.565) 0.00005	0.426 (0.241, 0.752) 0.00329
**P for trend**	< 0.00001	0.00011	0.00631

Model 1: adjusted for no covariates

Model 2: adjusted for variables of age, gender, race, education, marital status, and PIR

Model 3: adjusted for variables of age, gender, race, education, marital status, PIR, smoking, drinking, PA, CVD, cancer, hypertension, diabetes, depression, and hyperlipemia

Abbreviations: SVR, skeletal muscle mass to visceral fat area ratio; PIR, ratio of family income to poverty; CVD, cardiovascular disease; PA, physical activity; MET, metabolic equivalent

### Restricted cubic spline (RCS) analyses

**Fig. 4:**
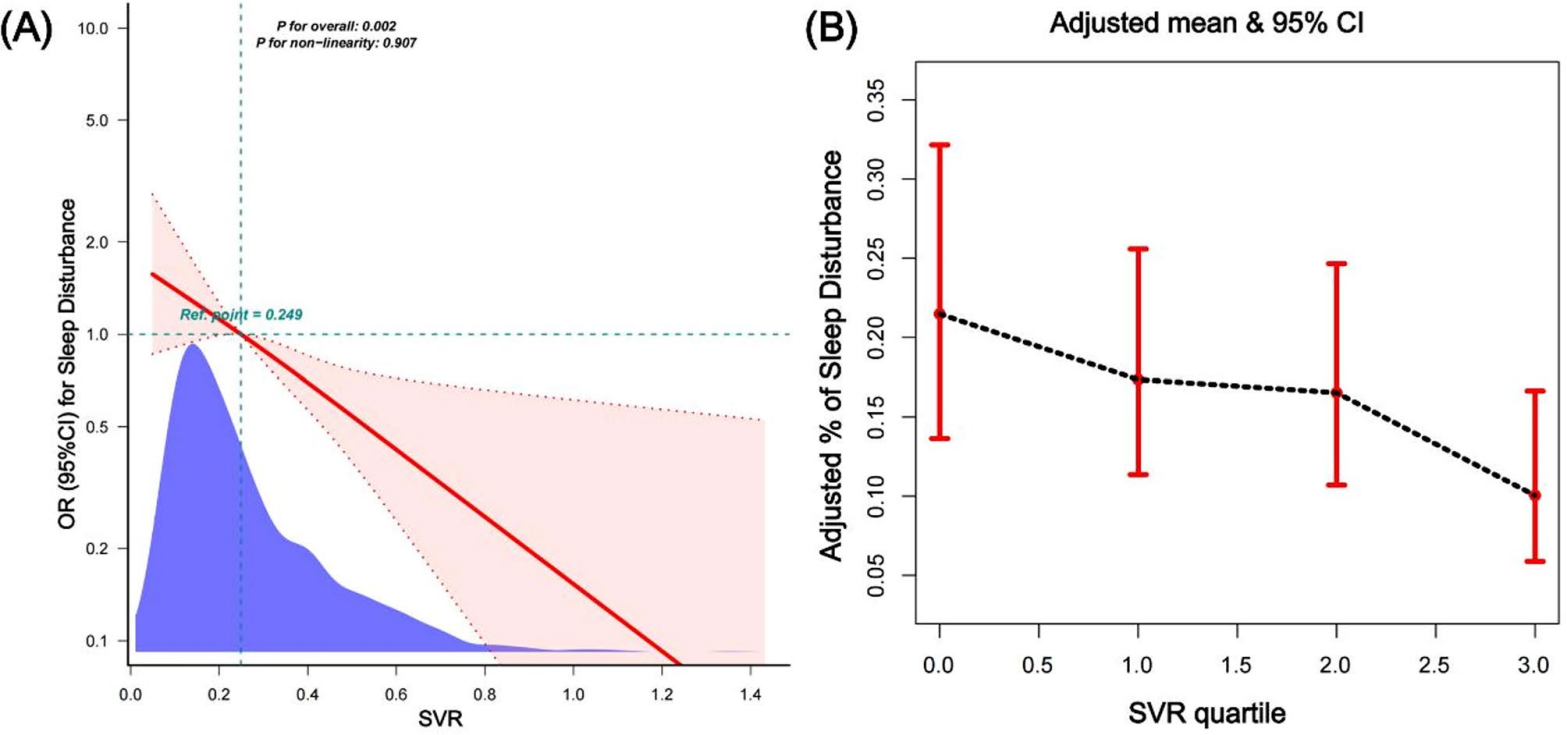
Identification of the association between SVR and sleep disturbance using RCS analysis. (**A**) The adjusted RCS analysis revealed the relationship between SVR (as continuous variable) and sleep disturbance. (**B**) The adjusted RCS analysis identified the association between SVR (as quadripartite variable) and sleep disturbance. Abbreviations: SVR, skeletal muscle mass to visceral fat area ratio; RCS, restricted cubic splines. Note: For higher SVR values (Fig. 4A, SVR > 1.0), confidence intervals widen significantly due to relatively sparse data in this range; results corresponding to extreme SVR values should be interpreted with caution

The adjusted RCS analysis was used to determine the potential nonlinear relationship between SVR and sleep disturbance. As shown in Fig. 4, a significantly negative and linear relationship between SVR and sleep disturbance was identified by RCS analysis (Fig. 4A) (P for overall = 0.002, P for nonlinearity = 0.907). When SVR was stratified into quartiles, RCS analysis revealed a stepwise reduction in sleep disturbance risk with higher SVR categories (Fig. 4B).

### Subgroup analyses and interaction analyses

Subgroup analyses identified significant heterogeneity in the association between SVR and sleep disturbance across strata of multiple variables (Table 4). Notably, this association varied substantially among subgroups delineated by gender, PIR, education level, smoking and drinking status, PA, and comorbidities including hypertension, hyperlipidemia, and asthma. A statistically significant inverse correlation was observed in females, individuals with lower PIR (< 3.5) or higher educational attainment, former/current smokers, mild alcohol consumers, and those with elevated PA levels. Additionally, the association remained significant in participants without hypertension or asthma, and also in individuals with depression or hyperlipidemia (all *P* < 0.05).

**Table 4:** Association between SVR and sleep disturbance in different subgroups

Characteristics	OR (95%CI) Pvalue	*P* for interaction
**Gender**		0.0142
Male	0.01 (0.00, 0.10) < 0.0001	
Female	0.42 (0.09, 0.89) 0.0067	
**Age categorical**		0.9814
<=40	0.09 (0.02, 0.47) 0.0039	
>40	0.05 (0.00, 0.58) 0.0161	
**Marital status**		0.2191
Married or living with a partner	0.16 (0.03, 0.92) 0.0401	
Living alone	0.04 (0.01, 0.39) 0.0048	
**PIR categorical**		0.7061
<=1.3	0.07 (0.00, 0.88) 0.0399	
1.3–3.5	0.04 (0.00, 0.33) 0.0030	
>3.5	0.40 (0.03, 4.72) 0.4662	
**Education**		0.9676
Less than high school	0.01 (0.00, 2.00) 0.0905	
high school or equivalent	0.03 (0.00, 0.51) 0.0148	
college or above	0.18 (0.03, 0.93) 0.0408	
**Smoking**		0.2773
Never	0.41 (0.06, 2.99) 0.3815	
Former	0.02 (0.00, 0.53) 0.0190	
Now	0.04 (0.00, 0.41) 0.0073	
**Drinking**		0.1171
Mild drinking	0.01 (0.00, 0.15) 0.0006	
Moderate drinking	0.22 (0.03, 1.64) 0.1406	
Heavy drinking	0.27 (0.02, 3.78) 0.3339	
**PA categorical**		0.8730
Low physical activity	0.06 (0.00, 1.52) 0.0879	
High physical activity	0.11 (0.02, 0.46) 0.0027	
**Hypertension**		0.2556
No	0.07 (0.01, 0.34) 0.0012	
Yes	0.23 (0.02, 2.30) 0.2086	
**Depression**		0.0390
No	0.00 (0.00, 0.01) 0.0006	
Yes	0.16 (0.04, 0.61) 0.0076	
**Hyperlipidemia**		0.2248
No	0.53 (0.11, 2.66) 0.4416	
Yes	0.01 (0.00, 0.06) < 0.0001	
**Asthma**		0.3403
No	0.06 (0.01, 0.27) 0.0003	
Yes	0.19 (0.01, 3.14) 0.2481	

Abbreviations: SVR, skeletal muscle mass to visceral fat area ratio; CVD, cardiovascular disease; PIR, ratio of family income to poverty; BMI, body mass index; PA, physical activity. Except for the stratifying variable, the variables adjusted in subgroup analyses were consistent with Model 3 in Table 3

**Fig. 5:**
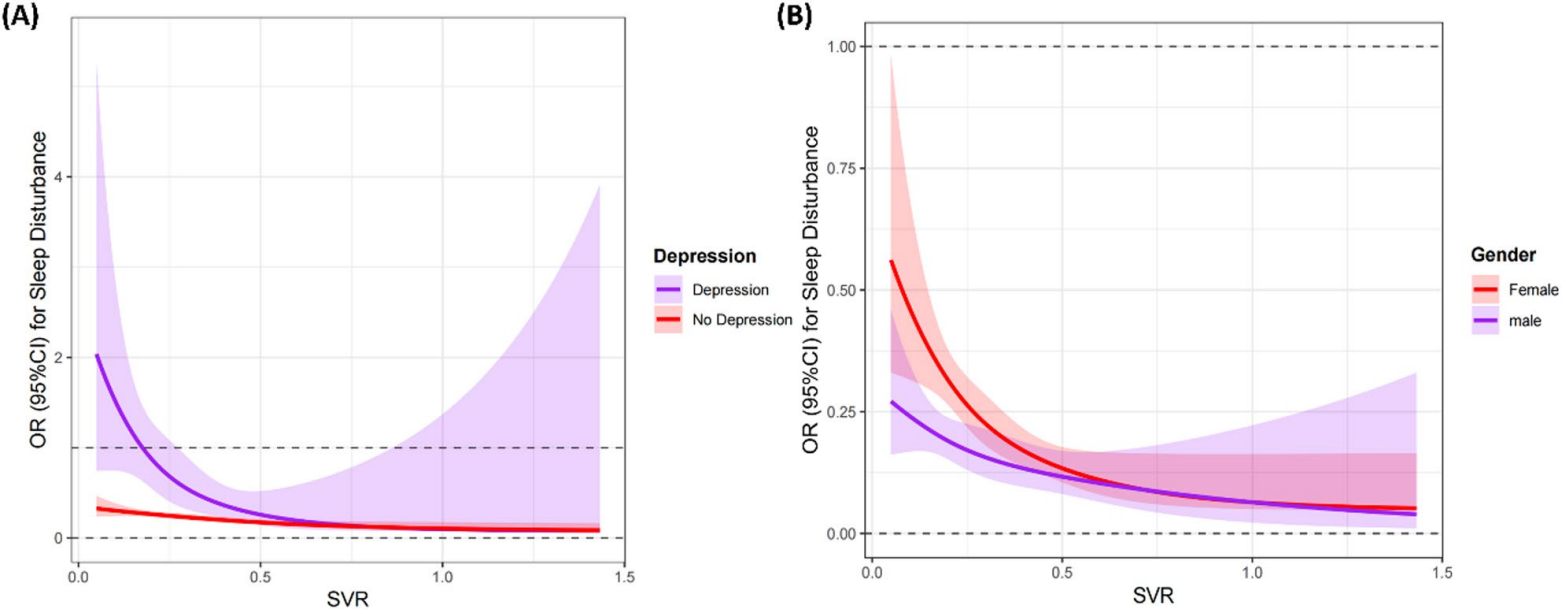
Stratified RCS analyses revealed the associations between SVR and sleep disturbance in different subgroups. (**A**) RCS curve analysis based on stratification of depression, (**B**) RCS curve analysis based on stratification of gender. The variables consistent with those in Table 3 (model 3) were adjusted during RCS analysis. Abbreviations: SVR, skeletal muscle mass to visceral fat area ratio; RCS, restricted cubic splines. Note: For higher SVR values (SVR > 1.0), confidence intervals widen significantly due to relatively sparse data in this range; results corresponding to extreme SVR values should be interpreted with caution

Interaction tests revealed that gender (*P* = 0.0142) and depression (*P* = 0.0390) were observed to interact with the association between SVR and sleep disturbance. Individuals who were males (OR = 0.01, 95% CI: 0.00-0.10) had relatively lower risk of sleep disturbance compared those who were females (OR = 0.42, 95% CI: 0.09–0.89) (Table 4). Individuals with depression (OR = 0.16, 95% CI: 0.04–0.61) showed a higher risk for sleep disturbance as opposed to those without depression (OR = 0.00, 95% CI: 0.00-0.01) (Table 4). These results were further verified by the findings from the stratified RCS analyses, which showed higher OR levels among females and depressed individuals (Fig. 5).

### The mediating role of AISI, CALLY, and PhenoAgeAccel in the association between SVR and sleep disturbance

To explore the mediating role of AISI, CALLY, and PhenoAgeAccel in the association between SVR and sleep disturbance, we performed mediation analysis. Prior to conducting this analysis, we first evaluated the correlation and multicollinearity among the three mediating variables. The absolute correlation coefficients between them ranged from 0.12 to 0.16, indicating weak correlations (|r| < 0.3; SFig. 2). After adjusting for other covariates, their variance inflation factors (VIFs) were 1.2, 1.27, and 1.29, respectively (STable 2). These results confirmed only slight collinearity—an issue that exerted minimal impact on the subsequent regression outcomes.

**Fig. 6:**
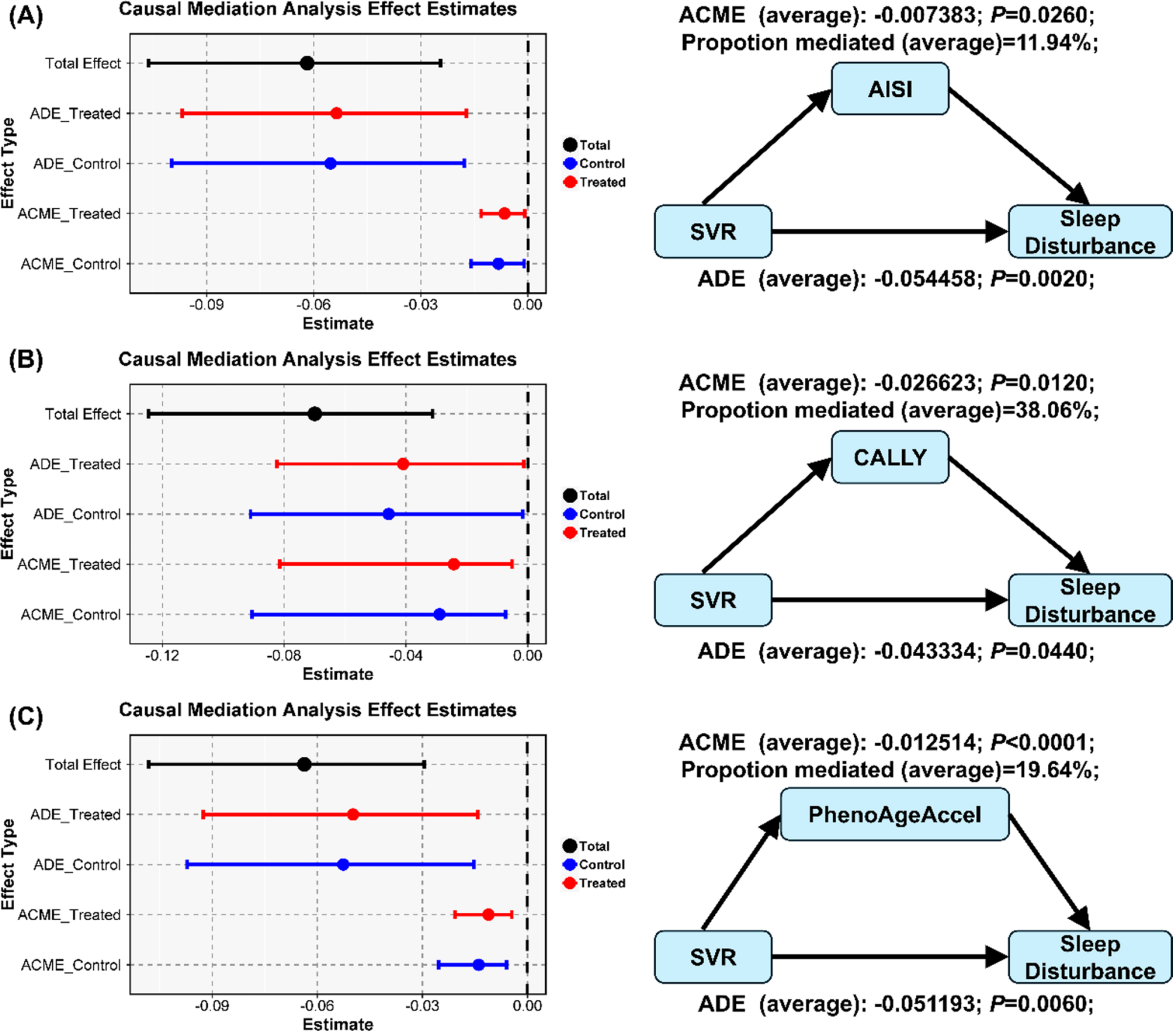
Inflammation and biological aging mediates the association between SVR and sleep disturbance. (**A**) AISI mediates the association between SVR and sleep disturbance; (**B**) CALLY mediates the association between SVR and sleep disturbance; (**C**) PhenoAgeAccel mediates the association between SVR and sleep disturbance. Abbreviations: SVR, skeletal muscle mass to visceral fat area ratio; AISI, aggregate index of systemic inflammation; CALLY, C-reactive Protein-Albumin-Lymphocyte Index; PhenoAgeAccel, Phenotypic age acceleration

In the primary single-mediator analyses (unadjusted for alternative mediators), AISI, CALLY, and PhenoAgeAccel all significantly partially mediated the effect of SVR on sleep disturbance (all P value < 0.05). The estimated indirect effects were as follows: AISI mediated 11.94% of the association (average causal mediation effect [ACME]=-0.007383, *P* = 0.0260ega), CALLY mediated 38.06% (ACME=-0.026623, *P* = 0.0120), and PhenoAgeAccel mediated 19.64% (ACME=-0.012514, *P* < 0.0001) (Fig. 6).

To verify the robustness of these findings and to evaluate the independent contribution of each mediator, we conducted sensitivity analyses in which each single-mediator model was additionally adjusted for the remaining mediator(s) as covariates. These models yielded indirect effects adjusted for the alternative mediators (i.e., conditional indirect effects). As shown in Fig. 6 and SFig. 3, the mediation percentages remained largely stable across model specifications: AISI (11.94% [unadjusted for other mediators] vs. 11.40% [adjusted for one mediator] vs. 10.64% [adjusted for two mediators]), CALLY (38.06% vs. 36.97% vs. 36.43%), and PhenoAgeAccel (19.64% vs. 19.33% vs. 15.99%). The minimal attenuation of these estimates indicates that each mediator exerts a robust and largely independent mediating effect in the relationship between SVR and sleep disturbance.

## Discussion

This study investigated the association between SVR and sleep disturbance in a nationally representative sample of 1,564 U.S. adults from the NHANES database. Our findings revealed a robust inverse association between SVR and sleep disturbance, with a linear negative dose-response relationship observed across SVR quartiles. Notably, this association was more pronounced in females and individuals with depression, suggesting potential effect modification by sex and mental health status. Furthermore, mediation analyses indicated that systemic inflammation (as indicated by AISI and CALLY) and biological aging (as reflected by PhenoAgeAccel) statistically explained a substantial portion of this association, providing preliminary insight into the underlying biological pathways. Collectively, these results position SVR as a potential protective factor against sleep disturbance and highlight the interplay between body composition, inflammation, and aging in sleep regulation.

Our study was the first one to elucidate the negative linear association between SVR and sleep disturbance. Our results demonstrated that lower SVR levels were associated with increased risk of developing sleep disturbance. This association can be understand from the following aspects. Individuals with a low SVR level indicates he/she got a significantly reduced muscle mass, increased visceral fat accumulation, or with both situations occurred simultaneously. These conditions have been proved to be associated with poor sleep quality in previous studies. Detailedly speaking, sarcopenia, low appendicular skeletal muscle mass (ASM) level, and reduced muscle strength were all associated with increased risk of sleep disorders among individuals with different health conditions. For example, evidence from previous study revealed that, in patients who suffered chronic liver diseases, decreased grip strength (GS), or the coexistence of reduced GS and skeletal muscle mass (SMM), was significantly associated with a higher Pittsburgh sleep quality index (PSQI-J) score (≥ 6 points) [67]. Similar findings were observed in patients with chronic obstructive pulmonary disease [68]. Moreover, among 318 aged outpatients with diabetes (≥ 65 years), both females (OR = 6.33, 95%CI: 1.91–20.97) and males (OR = 6.04, 95%CI: 1.71–21.36) with sarcopenia (assessed by the Japanese version of SARC-F) were found to link to increase ORs of developing sleep disorder [12]. Additionally, a systematic review based on middle-aged and aged individuals suggested a robust relationship between weak muscle strength and poor sleep quality/duration [13]. All these results have highlighted the critical role of reduced muscle mass in developing sleep disturbance. Amounting evidence has identified the relationship between fat accumulation, obesity-related indices, and sleep disturbance in previous studies. The results from a NHANES (2005–2018) study included 2,570 older people indicated that obese individuals got increased ORs of developing sleep disorder [69]. Similar findings from another study which revealed that each one-unit increase in body roundness index (BRI, an anthropometric indicator of obesity and visceral fat levels) was corresponding to a 13% higher risk of suffering sleep disorder (OR = 1.13, 95% CI: 1.09–1.16) [22]. These findings above provide strong evidence-based support for the results of this study and demonstrate the validity and reliability of our research findings. As a composite indicator integrating status of muscle loss and accumulation of visceral fat, SVR thus potentially represented a reliable and effective biomarker for assessing risk of sleep disturbance. Additionally, it should be noted that the confidence intervals (CIs) of the RCS curve for higher SVR values substantially widened. This phenomenon is primarily attributed to sparse data in the extreme SVR range—fewer observations in this segment reduce the precision of parameter estimates, leading to broader CIs. The characteristics of SVR distribution precisely confirm this inference, as they reveal a small number of samples (12/1564) when SVR is greater than 1. This may be the primary reason for prediction inaccuracies. Therefore, conclusions drawn from extreme SVR values (> 1) should be treated with caution: they may not be representative of the general trend and thus necessitate further validation using larger sample sizes that include more extreme SVR cases.

The results of the present study primarily delineate that sarcopenic obesity exhibits a strong association with an elevated risk of sleep disturbance. However, owing to the cross-sectional design of this research, the directionality of this association remains unconfirmed. Notably, a substantial body of evidence from prior studies underscores a complex, bidirectional relationship between sarcopenic obesity and sleep disturbance—these two conditions frequently co-occur, interact reciprocally, and exacerbate one another’s adverse effects [70, 71]. For one thing, obesity can induce respiratory sleep disorders (e.g., obstructive sleep apnea [OSA], obesity hypoventilation syndrome [OHS]) by perturbing the respiratory system, and may also precipitate non-respiratory sleep disorders (e.g., insomnia, restless legs syndrome [RLS]) via alternative mechanisms [70]. Research has demonstrated that augmented fat accumulation in the cervical and pharyngeal structures of obese individuals reduces the luminal volume of the airway and triggers airway collapse during sleep [72]. Concurrently, abdominal fat deposition diminishes lung volume, longitudinal tracheal traction, and upper airway wall tension, thereby contributing to pharyngeal airway collapse [73]. Additionally, the activity or responsiveness of upper airway dilator muscles is attenuated in obese patients, compromising their adaptive capacity to sustain airway patency during sleep [73]. Furthermore, alterations in the hormonal and cytokine profiles of obese individuals can elicit respiratory instability through pathways such as the impairment of neuroanatomical crosstalk [74]. All these mechanisms collectively disrupt respiratory function, culminating in respiratory sleep disturbance. Ultimately, obese individuals may also develop non-respiratory sleep disturbance via processes involving the dysregulation of iron homeostasis and disruptions to the limbic and nigrostriatal dopaminergic pathways [70]. For another, sleep disturbance can disturb the homeostasis of appetite-regulating hormones (e.g., leptin and ghrelin), elevate caloric intake, and reduce physical activity—factors that collectively induce energy imbalance, which in turn facilitates the onset and progression of obesity [71]. In conclusion, the correlation between obesity and an increased risk of sleep disturbance, as validated by the present study, aligns with the findings synthesized from prior research. Nevertheless, to date, no definitive consensus has been reached regarding the pathophysiological causal sequence of sarcopenic obesity and sleep disorders, which necessitates further in-depth investigation.

In this study, we found that there were significant gender differences in the association between SVR and sleep disturbance. Several key lines of evidence from previous studies may account for this phenomenon. Differences in sex hormone action, metabolic and inflammatory responses, and social behavior factors can all exert an effect on this association. Compared with females, males had higher levels of androgen, which were responsible for the synthesis of skeletal muscle protein and thus elevate SVR levels, contributing to a protective role of SVR against sleep disturbance [75]. Moreover, although females usually have less visceral fat than males [76, 77], it has shown a stronger association with metabolic health status and inflammatory markers, which may counteract the protective effect of SVR [78, 79]. Additionally, gender differences in stress response orchestrated by the hypothalamic-pituitary-adrenal (HPA) axis may also play a role in differing susceptibility to developing sleep disturbance [80]. Beyond the above factors, depression has been shown to exhibit a stronger association with sleep disturbance. A bidirectional relationship between depression and sleep disturbance has been identified in previous studies [81]. Various factors related to depression may contribute to sleep disturbance, including neurotransmitter imbalance, circadian rhythm dysregulation, hyperactivation of the HPA axis, structural and functional changes in the brain, inflammatory factors, metabolic abnormalities, anxiety, and nocturnal rumination [81], etc. It was estimated that approximately 90% of depressive patients complained of suffering sleep disturbance, such as insomnia, narcolepsy, and restless legs syndrome [81, 82]. Thus, our results were consistent with previous findings in this aspect. Taken together, these facts indicated that sex-related factors and depressive comorbidity should be taken into account when formulating targeted interventions to mitigate sleep disorders.

Our study found that indicators of inflammation (AISI and CALLY) and biological aging (PhenoAgeAccel) served as partial mediators in the association between SVR and sleep disturbance. Moreover, estimates from single‑mediator models unadjusted for alternative mediators and sensitivity analyses adjusting for the other mediators were consistent, further supporting the robustness of these findings. Specifically, the inverse association between SVR and AISI, CALLY, and PhenoAgeAccel, combined with the positive association between these mediators and sleep disturbance, suggests that higher SVR levels may help alleviate sleep disturbance by reducing systemic inflammation and slowing biological aging. These findings align with existing evidence demonstrating a bidirectional relationship of sleep with inflammation and accelerated aging. Prior studies have shown that sleep disturbance can promote the release of pro-inflammatory cytokines (e.g., IL-6, TNF, IL-1), which in turn further impair sleep regulation [83]. Similarly, accelerated biological aging and sleep abnormalities (e.g., earlier sleep onset, reduced nocturnal sleep duration, more frequent daytime naps, increased nighttime awakenings, and prolonged nocturnal wakefulness, and diminished slow wave sleep) has been shown to be closely associated, forming a vicious cycle through key mechanisms such as chronic systemic inflammation, oxidative stress, hypothalamic–pituitary–adrenal (HPA) axis activation, and circadian rhythm disturbance [84, 85]. These findings collectively highlight the important roles of inflammation and aging in the development of sleep disturbance. From a mechanistic perspective, these pathways may be particularly relevant in the context of SO, where lower SVR levels are commonly observed. In this condition, adipocyte hypertrophy and activation promote macrophage infiltration and dysregulated adipokine secretion, leading to chronic low-grade inflammation [86, 87]. Concurrently, impaired lipid metabolism disrupts mitochondrial function, reduces fatty acid β-oxidation, and increases reactive oxygen species, thereby creating a lipotoxic environment that accelerates the aging process [88, 89]. The interaction between these inflammatory and pro-aging mechanisms may heighten susceptibility to sleep disturbance. Taken together, the mechanistic interplay between inflammation and aging in SO mentioned-above provides a plausible biological explanation for our results: lower SVR levels, reflecting more severe SO, are associated with enhanced inflammatory activity and accelerate aging, which in turn increase the odds of sleep disturbance. However, it is noteworthy that the relationship between SVR, mediators, and sleep disturbance reflects associative patterns rather than causal links, given the cross-sectional design of this study. Thus, the temporal sequence of changes for these variables cannot be determined and should be investigated in future longitudinal and interventional studies.

Our study offers several key strengths. First, it utilizes a relatively large sample from the NHANES database, which provides sufficient data volume to support the application of diverse statistical methods and enhances the statistical robustness of the observed results. Second, through multivariable adjustment, quartile-based trend tests, restricted cubic spline curve analysis, and a variety of other statistical approaches, the robustness of the association between SVR and sleep disturbance was rigorously established. Third, our study identified a stronger association between SVR and sleep disturbance in females and individuals with depression, highlighting the need for targeted sex-stratified interventions and combined metabolic-psychological therapies.

Several limitations of this study should be acknowledged. First, certain variables—including self-reported sleep parameters, smoking status, alcohol consumption, CVD history, and physical activity—were derived from participant questionnaires, which may introduce potential bias into the results. Second, the cross-sectional design precludes establishing causal relationships between SVR and sleep disturbance; thus, longitudinal cohort studies are needed to validate these findings in the future. Third, the inability to extract data on adipose tissue-secreted factors from the NHANES database hindered our further analysis of their potential association with sleep disorders. Future studies could integrate multi-omics research strategies to explore differences in metabolomics, inflammatory profiles, and proteomics between healthy subjects and those with low SVR, thereby further investigating the underlying mechanisms by which sarcopenic obesity increases the risk of sleep disorders. Fourth, the cross-sectional design of the study means that the mediating roles of AISI, CALLY, and PhenoAgeAccel—which respectively reflect inflammatory status (AISI, CALLY) and aging acceleration (PhenoAgeAccel)—identified in this study are exploratory. Further longitudinal or interventional studies are thus needed to verify the temporal order of changes in these variables and confirm the definitive causal pathways underlying sleep disturbance related to lower SVR levels. Fifth, although we have selected and adjusted for covariates on the basis of statistical findings and key literature, we cannot rule out the possibility that other critical variables remain unaccounted for. Unmeasured factors—such as genetic susceptibility to sleep, psychosocial stress, noise interference, and personal living habits—may likewise influence the association between SVR and sleep disturbance. Sixth, the tool used in this study to assess sleep disturbance mainly captured the characteristics of sleep apnea. Thus, our findings mainly reflect the association between sarcopenic obesity and sleep apnea, while other sleep disorders, such as insomnia and restless legs syndrome, were likely underrepresented due to the limitations of the questionnaire. Consequently, the observed associations may not generalize to all types of sleep disturbance. Future studies should adopt more comprehensive sleep assessment methods, such as polysomnography combined with validated questionnaires targeting other sleep disorders, to validate and extend our findings.

## Conclusion

In summary, our study demonstrated a significant inverse linear association between SVR and sleep disturbance in U.S. adults. As a composite index integrating ASM and VFA, SVR offers a more comprehensive perspective on the relationship between body composition and sleep disorders. However, given the cross-sectional design of the present study, the causal mechanisms linking lower SVR to an elevated risk of sleep disturbance cannot be established. Accordingly, future longitudinal studies that incorporate multi-omics approaches are warranted to further elucidate these potential causal pathways.

## Supplementary Information

Below is the link to the electronic supplementary material.

Supplementary Material 1

Supplementary Material 2

Supplementary Material 3

Supplementary Material 4

## Data Availability

This study analyzed publicly available datasets, which can be accessed at the following link: https://wwwn.cdc.gov/nchs/nhanes/continuousnhanes/default.aspx.

## Abbreviations

SVR: Skeletal muscle mass to visceral fat area ratio
PIR: Ratio of family income to poverty
CVD: Cardiovascular disease
PA: Physical activity
MET: Metabolic equivalent

## References

[1] You Y, Chen Y, Fang W, Li X, Wang R, Liu J, et al. The association between sedentary behavior, exercise, and sleep disturbance: A mediation analysis of inflammatory biomarkers. Front Immunol. 2023;13:1080782. 10.3389/fimmu.2022.1080782. 36713451 10.3389/fimmu.2022.1080782 PMC9880546

[2] Léger D, Poursain B, Neubauer D, Uchiyama M. An international survey of sleeping problems in the general population. Curr Med Res Opin. 2008;24:307–17. 10.1185/030079907x253771. 18070379 10.1185/030079907x253771

[3] Hui SA, Grandner MA. Trouble Sleeping Associated With Lower Work Performance and Greater Health Care Costs: Longitudinal Data From Kansas State Employee Wellness Program. J Occup Environ Med. 2015;57:1031–8. 10.1097/JOM.0000000000000534. 10.1097/JOM.0000000000000534 PMC4610176 26461857

[4] Gangwisch JE, Heymsfield SB, Boden-Albala B, Buijs RM, Kreier F, Pickering TG, et al. Sleep duration as a risk factor for diabetes incidence in a large U.S. sample. Sleep. 2007;30:1667–73. 10.1093/sleep/30.12.1667. 18246976 10.1093/sleep/30.12.1667 PMC2276127

[5] Adamo D, Ruoppo E, Leuci S, Aria M, Amato M, Mignogna MD. Sleep disturbances, anxiety and depression in patients with oral lichen planus: a case-control study. J Eur Acad Dermatol Venereol. 2015;29:291–7. 10.1111/jdv.12525. 24754427 10.1111/jdv.12525

[6] St-Onge M-P, Grandner MA, Brown D, Conroy MB, Jean-Louis G, Coons M, et al. Sleep Duration and Quality: Impact on Lifestyle Behaviors and Cardiometabolic Health: A Scientific Statement From the American Heart Association. Circulation. 2016;134:e367–86. 10.1161/CIR.0000000000000444. 27647451 10.1161/CIR.0000000000000444 PMC5567876

[7] Moore M, Meltzer LJ. The sleepy adolescent: causes and consequences of sleepiness in teens. Paediatr Respir Rev. 2008;9:114–20. 10.1016/j.prrv.2008.01.001. quiz 120–1. 18513671 10.1016/j.prrv.2008.01.001

[8] Hershner SD, Chervin RD. Causes and consequences of sleepiness among college students. Nat Sci Sleep. 2014;6:73–84. 10.2147/NSS.S62907. 25018659 10.2147/NSS.S62907 PMC4075951

[9] Owens J, Adolescent Sleep Working Group, Committee on Adolescence. Insufficient sleep in adolescents and young adults: an update on causes and consequences. Pediatrics. 2014;134:e921–932. 10.1542/peds.2014-1696. 25157012 10.1542/peds.2014-1696 PMC8194472

[10] Millman RP, Working Group on Sleepiness in Adolescents/Young Adults, AAP Committee on Adolescence. Excessive sleepiness in adolescents and young adults: causes, consequences, and treatment strategies. Pediatrics. 2005;115:1774–86. 10.1542/peds.2005-0772. 15930245 10.1542/peds.2005-0772

[11] Liu K, Luo J, Chen Y, Li B, Tian Y, Wang X, et al. Association between sarcopenia and sleep disorders: a cross-sectional population based study. Front Nutr. 2024;11:1415743. 10.3389/fnut.2024.1415743. 38962441 10.3389/fnut.2024.1415743 PMC11220616

[12] Ida S, Kaneko R, Nagata H, Noguchi Y, Araki Y, Nakai M, et al. Association between sarcopenia and sleep disorder in older patients with diabetes. Geriatr Gerontol Int. 2019;19:399–403. 10.1111/ggi.13627. 30773802 10.1111/ggi.13627

[13] St APPSAKAP, Vs C. Association between muscle strength and sleep quality and duration among middle-aged and older adults: a systematic review. Eur Geriatr Med. 2021. 10.1007/s41999-020-00399-8. 12. 10.1007/s41999-020-00399-8 32974889

[14] Yang J, Jiang F, Yang M, Chen Z. Sarcopenia and nervous system disorders. J Neurol. 2022;269:5787–97. 10.1007/s00415-022-11268-8. 35829759 10.1007/s00415-022-11268-8

[15] Li X, He J, Sun Q. Sleep Duration and Sarcopenia: An Updated Systematic Review and Meta-Analysis. J Am Med Dir Assoc. 2023;24:1193–e12065. 10.1016/j.jamda.2023.04.032. 37295459 10.1016/j.jamda.2023.04.032

[16] Shibuki T, Motooka I, Saito H, Shinkai K, Kobayashi K, Ando H, et al. The association between sleep parameters and sarcopenia in Japanese community-dwelling older adults. Arch Gerontol Geriatr. 2023;109. 10.1016/j.archger.2023.104948. 10.1016/j.archger.2023.104948 36764202

[17] You Y, Chen Y, Zhang Q, Yv N, Niu Y, Cao Q. Muscle quality index is associated with trouble sleeping: a cross-sectional population based study. BMC Public Health. 2023;23. 10.1186/s12889-023-15411-6. 10.1186/s12889-023-15411-6 PMC10012435 36918831

[18] Lv X, Peng W, Jia B, Lin P, Yang Z. Longitudinal association of sleep duration with possible sarcopenia: evidence from CHARLS. BMJ Open. 2024;14:e079237. 10.1136/bmjopen-2023-079237. 38521528 10.1136/bmjopen-2023-079237 PMC10961493

[19] Chen L, Li Q, Huang X, Li Z. Association between sleep duration and possible sarcopenia in middle-aged and elderly Chinese individuals: evidence from the China health and retirement longitudinal study. BMC Geriatr. 2024;24:594. 10.1186/s12877-024-05168-x. 38992611 10.1186/s12877-024-05168-x PMC11241889

[20] Covassin N, Singh P, McCrady-Spitzer SK, St Louis EK, Calvin AD, Levine JA, et al. Effects of Experimental Sleep Restriction on Energy Intake, Energy Expenditure, and Visceral Obesity. J Am Coll Cardiol. 2022;79:1254–65. 10.1016/j.jacc.2022.01.038. 35361348 10.1016/j.jacc.2022.01.038 PMC9187217

[21] Chaput J-P, Bouchard C, Tremblay A. Change in sleep duration and visceral fat accumulation over 6 years in adults. Obes (Silver Spring). 2014;22:E9–12. 10.1002/oby.20701. 10.1002/oby.20701 24420871

[22] Gong H, Zhao Y. Association between body roundness index and sleep disorder: the mediating role of depression. BMC Psychiatry. 2025;25:212. 10.1186/s12888-025-06664-z. 40055626 10.1186/s12888-025-06664-z PMC11889924

[23] Xu X, Xu J, Zhang M. Association between metabolic score for visceral fat and obstructive sleep apnea: a cross-sectional study. Front Med. 2024;11. 10.3389/fmed.2024.1480717 10.3389/fmed.2024.1480717 PMC11669583 39726679

[24] Mazzuca E, Battaglia S, Marrone O, Marotta AM, Castrogiovanni A, Esquinas C, et al. Gender-specific anthropometric markers of adiposity, metabolic syndrome and visceral adiposity index (VAI) in patients with obstructive sleep apnea. J Sleep Res. 2014;23:13–21. 10.1111/jsr.12088. 24118617 10.1111/jsr.12088

[25] Behnoush AH, Bahiraie P, Shokri Varniab Z, Foroutani L, Khalaji A. Composite lipid indices in patients with obstructive sleep apnea: a systematic review and meta-analysis. Lipids Health Dis. 2023;22:84. 10.1186/s12944-023-01859-3. 37386562 10.1186/s12944-023-01859-3 PMC10308736

[26] Bikov A, Frent S, Reisz D, Negru A, Gaita L, Breban Schwarzkopf D, et al. Comparison of Composite Lipid Indices in Patients with Obstructive Sleep Apnoea. Nat Sci Sleep. 2022;14:1333–40. 10.2147/NSS.S361318. 35923809 10.2147/NSS.S361318 PMC9342428

[27] Zhou T, Chen S, Mao J, Zhu P, Yu X, Lin R. Association between obstructive sleep apnea and visceral adiposity index and lipid accumulation product: NHANES 2015–2018. Lipids Health Dis. 2024;23:100. 10.1186/s12944-024-02081-5. 38600516 10.1186/s12944-024-02081-5 PMC11005189

[28] Wei S, Nguyen TT, Zhang Y, Ryu D, Gariani K. Sarcopenic obesity: epidemiology, pathophysiology, cardiovascular disease, mortality, and management. Front Endocrinol (Lausanne). 2023;14:1185221. 10.3389/fendo.2023.1185221. 37455897 10.3389/fendo.2023.1185221 PMC10344359

[29] Wei L, Wang B, Wang Y. Low handgrip strength with asymmetry is associated with elevated all-cause mortality risk in older Chinese adults with abdominal obesity. PLoS ONE. 2024;19:e0306982. 10.1371/journal.pone.0306982. 39137193 10.1371/journal.pone.0306982 PMC11321545

[30] Zhang X, Ma N, Lin Q, Chen K, Zheng F, Wu J, et al. Body Roundness Index and All-Cause Mortality Among US Adults. JAMA Netw Open. 2024;7:e2415051. 10.1001/jamanetworkopen.2024.15051. 38837158 10.1001/jamanetworkopen.2024.15051 PMC11154161

[31] Ma J, Xiu R, Li H, Zheng X. Associations between sarcopenic obesity and risk of cardiovascular disease: a population-based cohort study among middle-aged and older adults using the CHARLS. Clin Nutr. 2024;43. 10.1016/j.clnu.2024.02.002. 10.1016/j.clnu.2024.02.002 38350287

[32] Ye C, Chen G, Huang W, Liu Y. Association between skeletal muscle mass to visceral fat area ratio and depression: a cross-sectional study based on the National Health and Nutrition Examination Survey. J Affect Disord. 2025;372:314–23. 10.1016/j.jad.2024.12.041. 39667703 10.1016/j.jad.2024.12.041

[33] Kolb H. Obese visceral fat tissue inflammation: from protective to detrimental? BMC Med. 2022;20:494. 10.1186/s12916-022-02672-y. 36575472 10.1186/s12916-022-02672-y PMC9795790

[34] Batsis JA, Villareal DT. Sarcopenic obesity in older adults: aetiology, epidemiology and treatment strategies. Nat Rev Endocrinol. 2018;14:513–37. 10.1038/s41574-018-0062-9. 30065268 10.1038/s41574-018-0062-9 PMC6241236

[35] Mai Z, Chen Y, Mao H, Wang L. Association between the skeletal muscle mass to visceral fat area ratio and metabolic dysfunction-associated fatty liver disease: A cross-sectional study of NHANES 2017–2018. J Diabetes. 2024;16:e13569. 10.1111/1753-0407.13569. 38751375 10.1111/1753-0407.13569 PMC11096813

[36] Xing M, Ni Y, Zhang Y, Zhao X, Yu X. The relationship between skeletal muscle mass to visceral fat area ratio and metabolic dysfunction-associated fatty liver disease subtypes in middle-aged and elderly population: a single-center retrospective study. Front Nutr. 2023;10:1246157. 10.3389/fnut.2023.1246157. 38024359 10.3389/fnut.2023.1246157 PMC10663359

[37] Wang Q, Zheng D, Liu J, Fang L, Li Q. Skeletal muscle mass to visceral fat area ratio is an important determinant associated with type 2 diabetes and metabolic syndrome. Diabetes Metab Syndr Obes. 2019;12:1399–407. 10.2147/DMSO.S211529. 31616170 10.2147/DMSO.S211529 PMC6698596

[38] Low S, Ng TP, Goh KS, Moh A, Khoo J, Ang K, et al. Reduced skeletal muscle mass to visceral fat area ratio is independently associated with reduced cognitive function in type 2 diabetes mellitus. J Diabetes Complications. 2024;38:108672. 10.1016/j.jdiacomp.2023.108672. 38183854 10.1016/j.jdiacomp.2023.108672

[39] Ma Y-C, Li X-P, Lin X-Y, Zhang K-X, Leng J-Y. Role of immunity and inflammation in sarcopenic obesity. J Nutr Biochem. 2025;146:110077. 10.1016/j.jnutbio.2025.110077. 40840641 10.1016/j.jnutbio.2025.110077

[40] Lu W, Feng W, Lai J, Yuan D, Xiao W, Li Y. Role of adipokines in sarcopenia. Chin Med J (Engl). 2023;136:1794–804. 10.1097/CM9.0000000000002255. 37442757 10.1097/CM9.0000000000002255 PMC10406092

[41] Choi W, Woo GH, Kwon T-H, Jeon J-H. Obesity-Driven Metabolic Disorders: The Interplay of Inflammation and Mitochondrial Dysfunction. Int J Mol Sci. 2025;26:9715. 10.3390/ijms26199715. 41096980 10.3390/ijms26199715 PMC12525337

[42] Li C-W, Yu K, Shyh-Chang N, Jiang Z, Liu T, Ma S, et al. Pathogenesis of sarcopenia and the relationship with fat mass: descriptive review. J Cachexia Sarcopenia Muscle. 2022;13:781–94. 10.1002/jcsm.12901. 35106971 10.1002/jcsm.12901 PMC8977978

[43] Thangarajan R, Rammohan S, Ghosh M, Teja SA, Bakthavatchalam P, Swagatika S, et al. Circadian Misalignment and Inflammasome Dynamics: A Molecular Bridge Between Sleep and Inflammation. Chronobiol Med. 2026;8:16–31. 10.33069/cim.2025.0068.

[44] Hagag L. Circadian rhythm disturbances and immune system. Instituţia Publică Universitatea de Stat de Medicină şi Farmacie „Nicolae Testemiţanu din Republica Moldova; 2024. https://doi.org/10/28770.

[45] CDC. National Health and Nutrition Examination Survey. National Health and Nutrition Examination Survey. 2025. https://www.cdc.gov/nchs/nhanes/index.html. Accessed 2 Apr 2025.

[46] Wen Q, Wang Q, Yang H. The association between epilepsy and sleep disturbance in US adults: the mediating effect of depression. BMC Public Health. 2024;24:2412. 10.1186/s12889-024-19898-5. 39232706 10.1186/s12889-024-19898-5 PMC11375921

[47] Centers for Disease Control and Prevention. National Health and Nutrition Examination Survey. 2017–2018 Sleep Disorder Data. https://wwwn.cdc.gov/Nchs/Data/Nhanes/Public/2017/DataFiles/SLQ_J.htm. Accessed 2 Apr 2025.

[48] Duan J, Chen J, Xiang Z. The U-shape relationship between the aggregate index of systemic inflammation and depression in American adults: A cross-sectional study. J Affect Disord. 2025;380:270–8. 10.1016/j.jad.2025.03.139. 40147607 10.1016/j.jad.2025.03.139

[49] Xu Z, Tang J, Xin chen, Jin Y, Zhang H, Liang R. Associations of C-reactive protein-albumin-lymphocyte (CALLY) index with cardiorenal syndrome: Insights from a population-based study. Heliyon. 2024;10:e37197. 10.1016/j.heliyon.2024.e37197. 39296012 10.1016/j.heliyon.2024.e37197 PMC11408039

[50] Levine ME, Lu AT, Quach A, Chen BH, Assimes TL, Bandinelli S, et al. An epigenetic biomarker of aging for lifespan and healthspan. Aging. 2018;10:573–91. 10.18632/aging.101414. 29676998 10.18632/aging.101414 PMC5940111

[51] Yang Q, Zhu X, Zhang L, Luo F. Dyslipidemia and aging: the non-linear association between atherogenic index of plasma (AIP) and aging acceleration. Cardiovasc Diabetol. 2025;24:181. 10.1186/s12933-025-02695-8. 40281579 10.1186/s12933-025-02695-8 PMC12023499

[52] Dai W, Zhang D, Wei Z, Liu P, Yang Q, Zhang L, et al. Whether weekend warriors (WWs) achieve equivalent benefits in lipid accumulation products (LAP) reduction as other leisure-time physical activity patterns? Results from a population-based analysis of NHANES 2007–2018. BMC Public Health. 2024;24:1550. 10.1186/s12889-024-19070-z. 38853276 10.1186/s12889-024-19070-z PMC11163723

[53] Ly P. Association between triglyceride glucose index and biological aging in U.S. adults: National Health and Nutrition Examination Survey. Cardiovasc Diabetol. 2025;24. 10.1186/s12933-025-02631-w. 10.1186/s12933-025-02631-w PMC11871761 40022176

[54] Xu W, Zhang L, Yang Q, Cao Y, Rao R, Lv L, et al. Associations of prognostic nutritional index with cardiovascular all-cause mortality among CVD patients with diabetes or prediabetes: evidence from the NHANES 2005–2018. Front Immunol. 2025;16. 10.3389/fimmu.2025.1518295. 10.3389/fimmu.2025.1518295 PMC11860081 40013151

[55] Hou W, Chen S, Zhu C, Gu Y, Zhu L, Zhou Z. Associations between smoke exposure and osteoporosis or osteopenia in a US NHANES population of elderly individuals. Front Endocrinol (Lausanne). 2023;14:1074574. 10.3389/fendo.2023.1074574. 36817605 10.3389/fendo.2023.1074574 PMC9935577

[56] Zeng G, You D, Ye L, Wu Y, Shi H, Lin J, et al. n-3 PUFA poor seafood consumption is associated with higher risk of gout, whereas n-3 PUFA rich seafood is not: NHANES 2007–2016. Front Nutr. 2023;10:1075877. 10.3389/fnut.2023.1075877. 37081920 10.3389/fnut.2023.1075877 PMC10110868

[57] Xue H, Zou Y, Yang Q, Zhang Z, Zhang J, Wei X, et al. The association between different physical activity (PA) patterns and cardiometabolic index (CMI) in US adult population from NHANES (2007–2016). Heliyon. 2024;10:e28792. 10.1016/j.heliyon.2024.e28792. 38586407 10.1016/j.heliyon.2024.e28792 PMC10998206

[58] Tao X, Xu X, Xu Y, Yang Q, Yang T, Zhou X, et al. Association between physical activity and visceral adiposity index (VAI) in U.S. population with overweight or obesity: a cross-sectional study. BMC Public Health. 2024;24:2314. 10.1186/s12889-024-19810-1. 39187794 10.1186/s12889-024-19810-1 PMC11348595

[59] MacGregor KA, Gallagher IJ, Moran CN. Relationship Between Insulin Sensitivity and Menstrual Cycle Is Modified by BMI, Fitness, and Physical Activity in NHANES. J Clin Endocrinol Metab. 2021;106:2979–90. 10.1210/clinem/dgab415. 34111293 10.1210/clinem/dgab415 PMC8475204

[60] Leavitt MO. 2008 Physical Activity Guidelines for Americans.

[61] Cai Y, Chen M, Zhai W, Wang C. Interaction between trouble sleeping and depression on hypertension in the NHANES 2005–2018. BMC Public Health. 2022;22:481. 10.1186/s12889-022-12942-2. 35277151 10.1186/s12889-022-12942-2 PMC8917766

[62] Zhang Q, Xiao S, Jiao X, Shen Y. The triglyceride-glucose index is a predictor for cardiovascular and all-cause mortality in CVD patients with diabetes or pre-diabetes: evidence from NHANES 2001–2018. Cardiovasc Diabetol. 2023;22:279. 10.1186/s12933-023-02030-z. 37848879 10.1186/s12933-023-02030-z PMC10583314

[63] Smagula SF, Zhang G, Gujral S, Covassin N, Li J, Taylor WD, et al. Association of 24-Hour Activity Pattern Phenotypes With Depression Symptoms and Cognitive Performance in Aging. JAMA Psychiatry. 2022;79:1023–31. 10.1001/jamapsychiatry.2022.2573. 36044201 10.1001/jamapsychiatry.2022.2573 PMC9434485

[64] Wang G, Fang L, Chen Y, Ma Y, Zhao H, Wu Y, et al. Association between exposure to mixture of heavy metals and hyperlipidemia risk among U.S. adults: A cross-sectional study. Chemosphere. 2023;344:140334. 10.1016/j.chemosphere.2023.140334. 37788750 10.1016/j.chemosphere.2023.140334

[65] James G, Witten D, Hastie T, Tibshirani R. An Introduction to Statistical Learning: with Applications. New York, NY: Springer US; 2021. 10.1007/978-1-0716-1418-1.

[66] Zheng Y, Liu W, Zhu X, Xu M, Lin B, Bai Y. Associations of dietary inflammation index and composite dietary antioxidant index with preserved ratio impaired spirometry in US adults and the mediating roles of triglyceride-glucose index: NHANES 2007–2012. Redox Biol. 2024;76:103334. 10.1016/j.redox.2024.103334. 39217849 10.1016/j.redox.2024.103334 PMC11402638

[67] Nishikawa H, Enomoto H, Yoh K, Iwata Y, Sakai Y, Kishino K, et al. Effect of Sarcopenia on Sleep Disturbance in Patients with Chronic Liver Diseases. J Clin Med. 2018;8:16. 10.3390/jcm8010016. 30583494 10.3390/jcm8010016 PMC6352199

[68] Dala Pola D, Maia T, Moraes E, Ogochi L, Mesas A, Pitta F. Sarcopenia and sleep in individuals with chronic obstructive pulmonary disease. Sleep Breath. 2024;28:2557–63. 10.1007/s11325-024-03126-w. 39287720 10.1007/s11325-024-03126-w

[69] Wang W, Chen Z, Zhang W, Yuan R, Sun Y, Yao Q, et al. Association between obesity and sleep disorder in the elderly: evidence from NHANES 2005–2018. Front Nutr. 2024;11:1401477. 10.3389/fnut.2024.1401477. 39267860 10.3389/fnut.2024.1401477 PMC11390407

[70] Figorilli M, Velluzzi F, Redolfi S. Obesity and sleep disorders: A bidirectional relationship. Nutr Metabolism Cardiovasc Dis. 2025;35:104014. 10.1016/j.numecd.2025.104014. 10.1016/j.numecd.2025.104014 40180826

[71] Rodrigues GD, Fiorelli EM, Furlan L, Montano N, Tobaldini E. Obesity and sleep disturbances: The chicken or the egg question. Eur J Intern Med. 2021;92:11–6. 10.1016/j.ejim.2021.04.017. 33994249 10.1016/j.ejim.2021.04.017

[72] Davies RJ, Stradling JR. The relationship between neck circumference, radiographic pharyngeal anatomy, and the obstructive sleep apnoea syndrome. Eur Respir J. 1990;3:509–14. 2376247

[73] Schwartz AR, Patil SP, Laffan AM, Polotsky V, Schneider H, Smith PL. Obesity and obstructive sleep apnea: pathogenic mechanisms and therapeutic approaches. Proc Am Thorac Soc. 2008;5:185–92. 10.1513/pats.200708-137MG. 18250211 10.1513/pats.200708-137MG PMC2645252

[74] Wellman A, Jordan AS, Malhotra A, Fogel RB, Katz ES, Schory K, et al. Ventilatory control and airway anatomy in obstructive sleep apnea. Am J Respir Crit Care Med. 2004;170:1225–32. 10.1164/rccm.200404-510OC. 15317668 10.1164/rccm.200404-510OC PMC3861244

[75] Sheffield-Moore M. Androgens and the control of skeletal muscle protein synthesis. Ann Med. 2000;32:181–6. 10.3109/07853890008998825. 10821325 10.3109/07853890008998825

[76] Ludescher B, Najib A, Baar S, Machann J, Thamer C, Schick F, et al. Gender specific correlations of adrenal gland size and body fat distribution: a whole body MRI study. Horm Metab Res. 2007;39:515–8. 10.1055/s-2007-982518. 17611905 10.1055/s-2007-982518

[77] Beasley LE, Koster A, Newman AB, Javaid MK, Ferrucci L, Kritchevsky SB, et al. Inflammation and race and gender differences in computerized tomography-measured adipose depots. Obes (Silver Spring). 2009;17:1062–9. 10.1038/oby.2008.627. 10.1038/oby.2008.627 PMC3268118 19165157

[78] Zhang W, Su X, Liu S, Yue T, Tu Z, Zhang H, et al. Age-specific and sex-specific associations of visceral adipose tissue with metabolic health status and cardiovascular disease risk. Acta Diabetol. 2025. 10.1007/s00592-025-02447-w. 39792170 10.1007/s00592-025-02447-w

[79] Cartier A, Côté M, Lemieux I, Pérusse L, Tremblay A, Bouchard C, et al. Sex differences in inflammatory markers: what is the contribution of visceral adiposity? Am J Clin Nutr. 2009;89:1307–14. 10.3945/ajcn.2008.27030. 19297456 10.3945/ajcn.2008.27030

[80] Brivio E, Kos A, Ulivi AF, Karamihalev S, Ressle A, Stoffel R, et al. Sex shapes cell-type-specific transcriptional signatures of stress exposure in the mouse hypothalamus. Cell Rep. 2023;42. 10.1016/j.celrep.2023.112874. 10.1016/j.celrep.2023.112874 37516966

[81] Fang H, Tu S, Sheng J, Shao A. Depression in sleep disturbance: A review on a bidirectional relationship, mechanisms and treatment. J Cell Mol Med. 2019;23:2324–32. 10.1111/jcmm.14170. 30734486 10.1111/jcmm.14170 PMC6433686

[82] Tsuno N, Besset A, Ritchie K. Sleep and depression. J Clin Psychiatry. 2005;66:1254–69. 10.4088/jcp.v66n1008. 16259539 10.4088/jcp.v66n1008

[83] Veler H. Sleep and Inflammation: Bidirectional Relationship. Sleep Med Clin. 2023;18:213–8. 10.1016/j.jsmc.2023.02.003. 37120163 10.1016/j.jsmc.2023.02.003

[84] Gadodia R, Nandamuru D, Akberzie W, Kataria L. Sleep Disorders and Aging in Women. Sleep Med Clin. 2023;18:545–57. 10.1016/j.jsmc.2023.06.017. 38501526 10.1016/j.jsmc.2023.06.017

[85] Li J, Vitiello MV, Gooneratne NS. Sleep in Normal Aging. Sleep Med Clin. 2018;13:1–11. 10.1016/j.jsmc.2017.09.001. 29412976 10.1016/j.jsmc.2017.09.001 PMC5841578

[86] Meiliana A, Dewi NM, Defi IR, Rosdianto AM, Qiantori AA, Wijaya A. Sarcopenic Obesity: The Underlying Molecular Pathophysiology and Prospect Therapies. Indonesian Biomedical J. 2024;16:292–308. 10.18585/inabj.v16i4.3176.

[87] Nishikawa H, Fukunishi S, Asai A, Yokohama K, Nishiguchi S, Higuchi K. Pathophysiology and mechanisms of primary sarcopenia (Review). Int J Mol Med. 2021;48:1–8. 10.3892/ijmm.2021.4989. 10.3892/ijmm.2021.4989 34184088

[88] Bang S, Choi S-H, Jeong SM. Beyond Bioenergetics: Emerging Roles of Mitochondrial Fatty Acid Oxidation in Stress Response and Aging. Cells. 2025;14:1956. 10.3390/cells14241956. 41439977 10.3390/cells14241956 PMC12732221

[89] Ademowo OS, Dias HKI, Burton DGA, Griffiths HR. Lipid (per) oxidation in mitochondria: an emerging target in the ageing process? Biogerontology. 2017;18:859–79. 10.1007/s10522-017-9710-z. 28540446 10.1007/s10522-017-9710-z PMC5684309

