# Supplementary material for: The inverse association between skeletal muscle mass to visceral fat ratio (SVR) and sleep disturbance: the mediating role of inflammation and aging acceleration
Source: BMC Psychiatry. 2026 Jun 5;26:582. doi: 10.1186/s12888-026-08248-x (PMC13428428; doi:10.1186/s12888-026-08248-x)
Supplement: Supplementary file 1 — Supplementary Material 1 [file 12888_2026_8248_MOESM1_ESM.docx]

**STable 1. Definition of sleep quality score**

| **Sleep factors** | **Sleep condition** | **Sleep score** |
| --- | --- | --- |
| Sleep duration (h) | <7 | 0 |
|  | 7–9 | 1 |
|  | >9 | 0 |
| Trouble sleeping | No | 1 |
|  | Yes | 0 |
| Snoring | Never | 1 |
|  | Rarely/occasionally/frequently | 0 |
| Excessive daytime sleep | Never/rarely | 1 |
|  | Rarely/sometimes/often/almost always | 0 |
| Sleep apnea symptoms | Never | 1 |
|  | Rarely/occasionally/frequently | 0 |
| Sleep quality score |  | 0–5 |

**Note:**

1. For snoring, rarely indicates 1-2 nights a week, occasionally indicates 3-4 nights a week, and frequently means 5 or more nights a week.
2. For excessive daytime sleep, rarely indicates 1 time a month, sometimes means 2-4 times a month, often means 5-15 times a month, and almost always means 16-30 times a month.
3. For sleep apnea symptoms, rarely means 1-2 nights a week, occasionally means 3-4 nights a week, and frequently means 5 or more nights a week.

**STable 2. Multicollinearity detection for variables of AISI, CALLY and PhenoAgeAccel**

| **Variables** | **VIF** |
| --- | --- |
| AISI | 1.20 |
| CALLY | 1.27 |
| PhenoAgeAccel | 1.29 |

**Abbreviations:** AISI, aggregate index of systemic inflammation; CALLY, C-reactive Protein-Albumin-Lymphocyte Index; PhenoAgeAccel, Phenotypic age acceleration; VIF, variance inflation factor.


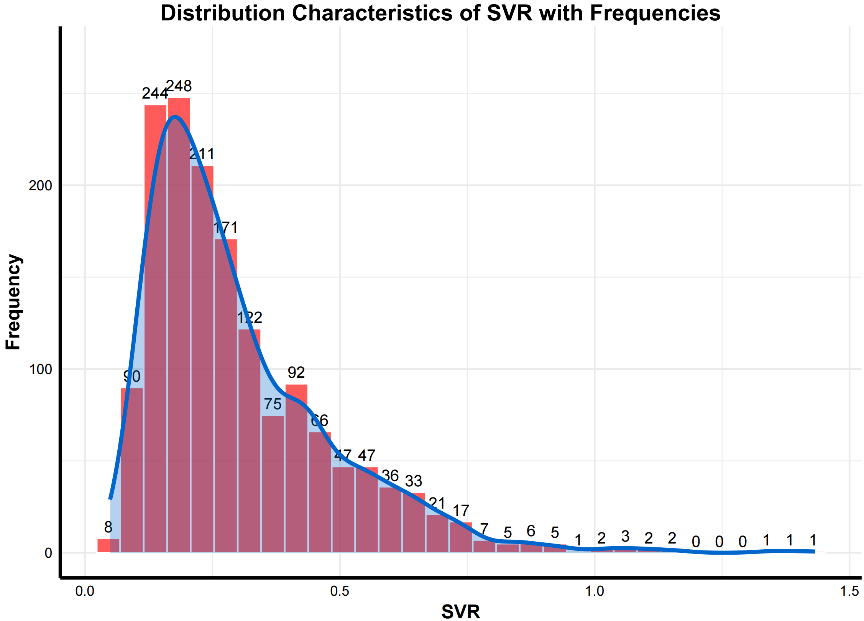


**SFigure 1**. **The distribution characteristics of SVR.** The red bars represent the frequency counts corresponding to different SVR values, and the blue line reflects the changing trend of the frequency distribution.


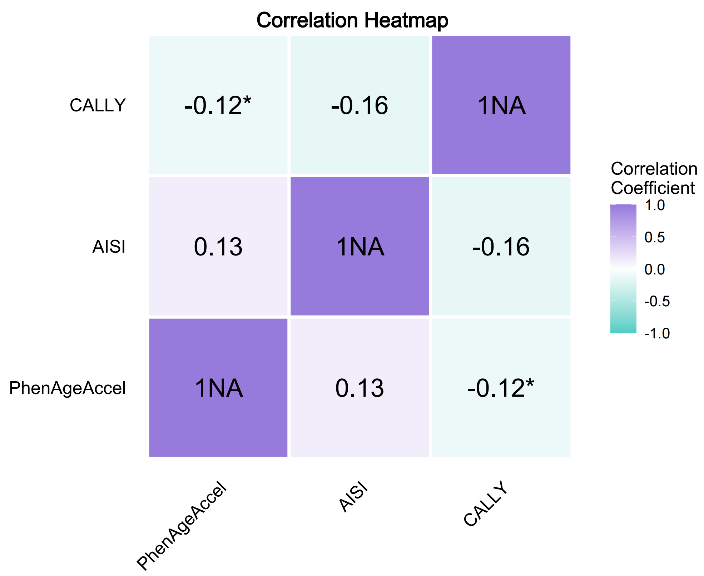


**SFigure 2. Pearson correlation heatmap for variables SVR, CALLY, AISI, and PhenAgeAccel.** The numbers in the cells represent correlation coefficients, and the "*" symbol indicates whether there is statistical significance. The right color scale shows correlation direction/strength. "1NA" denotes variable self-association.


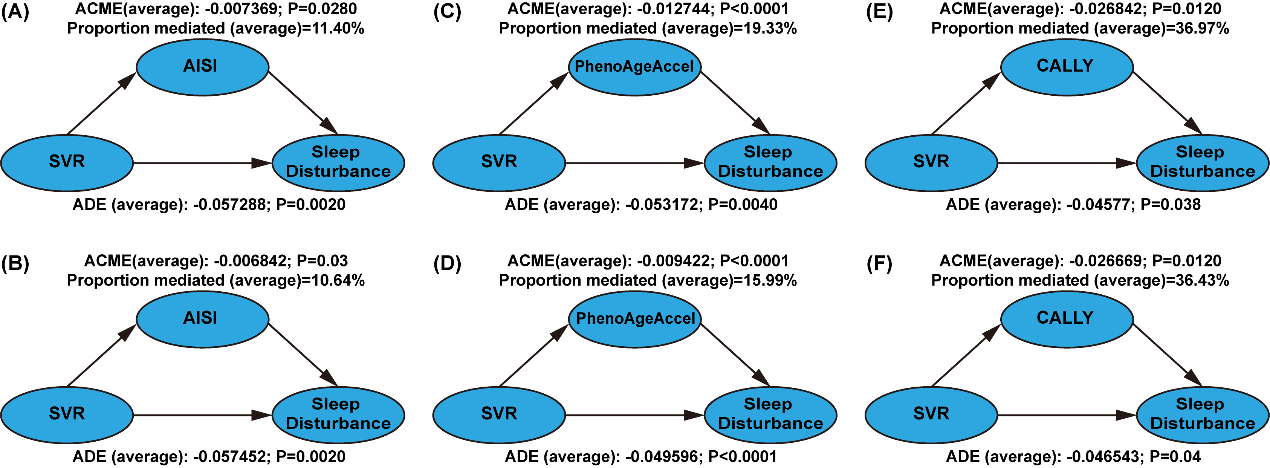


**SFigure 3. Sensitivity analyses of mediating effect.** (A) Further adjusted for PhenoAgeAccel, based on the adjusted covariates from Model 3 in Table 2. (B) Further adjusted for PhenoAgeAccel and CALLY, based on the adjusted covariates from Model 3 in Table 2. (C) Further adjusted for AISI, based on the adjusted covariates from Model 3 in Table 2. (D) Further adjusted for AISI and CALLY, based on the adjusted covariates from Model 3 in Table 2.(E) Further adjusted for AISI, based on the adjusted covariates from Model 3 in Table 2. (F) Further adjusted for AISI and PhenoAgeAccel, based on the adjusted covariates from Model 3 in Table 2.
