# Supplementary figures and images for: The inverse association between skeletal muscle mass to visceral fat ratio (SVR) and sleep disturbance: the mediating role of inflammation and aging acceleration
Source: BMC Psychiatry. 2026 Jun 5;26:582. doi: 10.1186/s12888-026-08248-x (PMC13428428; doi:10.1186/s12888-026-08248-x)

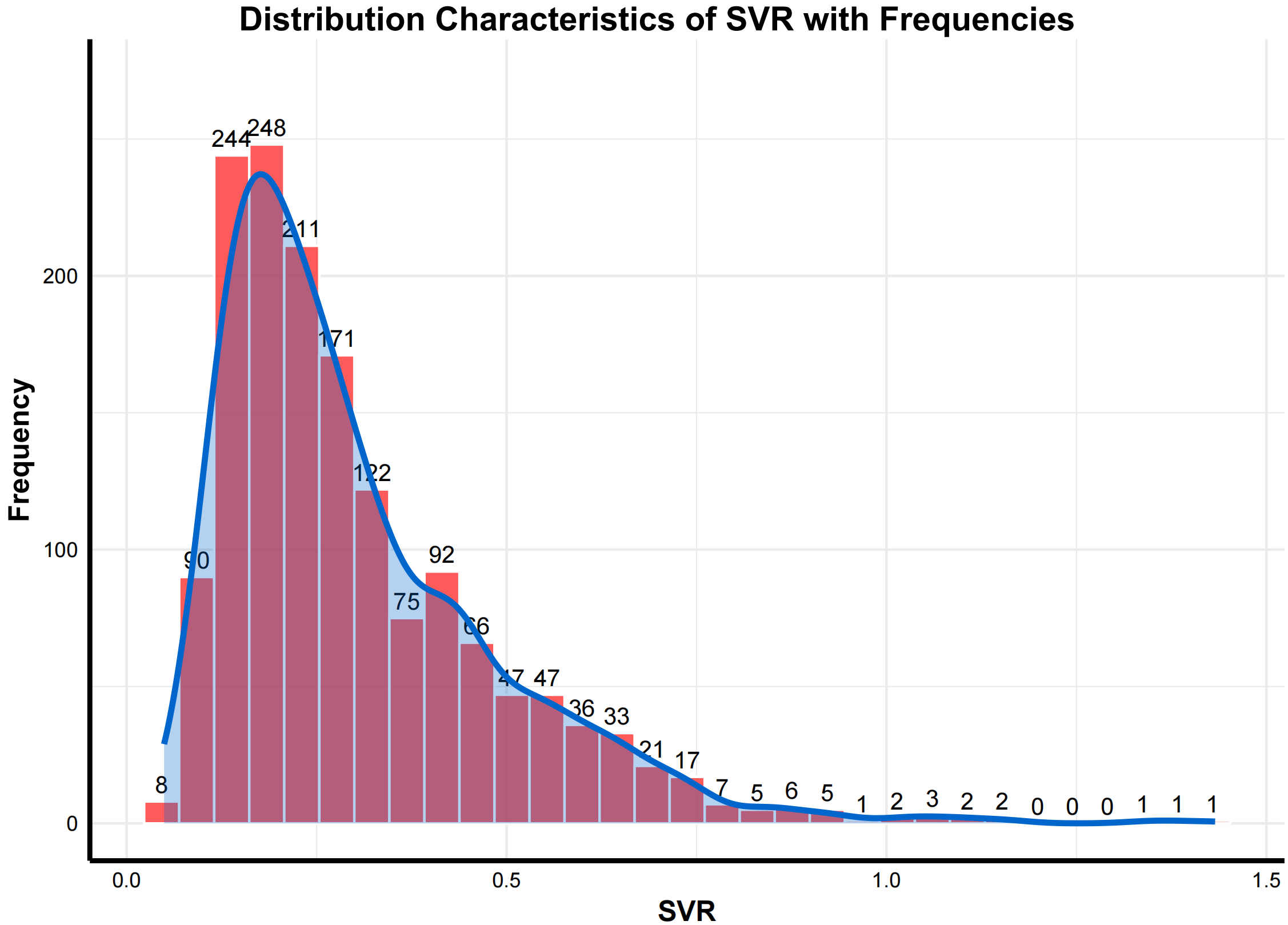

Supplement: Supplementary file 2 — Supplementary Material 2 [file 12888_2026_8248_MOESM2_ESM.tif]

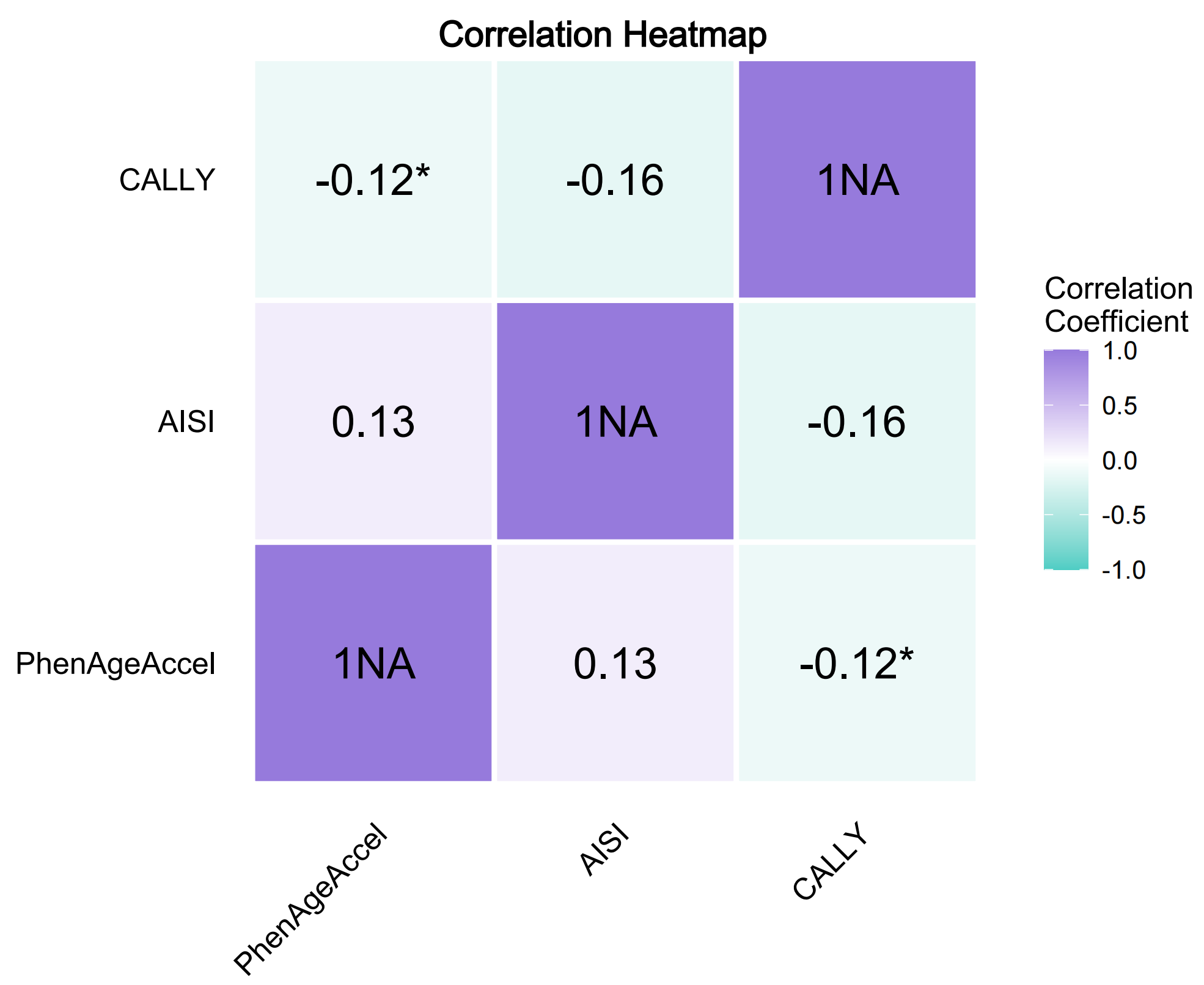

Supplement: Supplementary file 3 — Supplementary Material 3 [file 12888_2026_8248_MOESM3_ESM.tif]

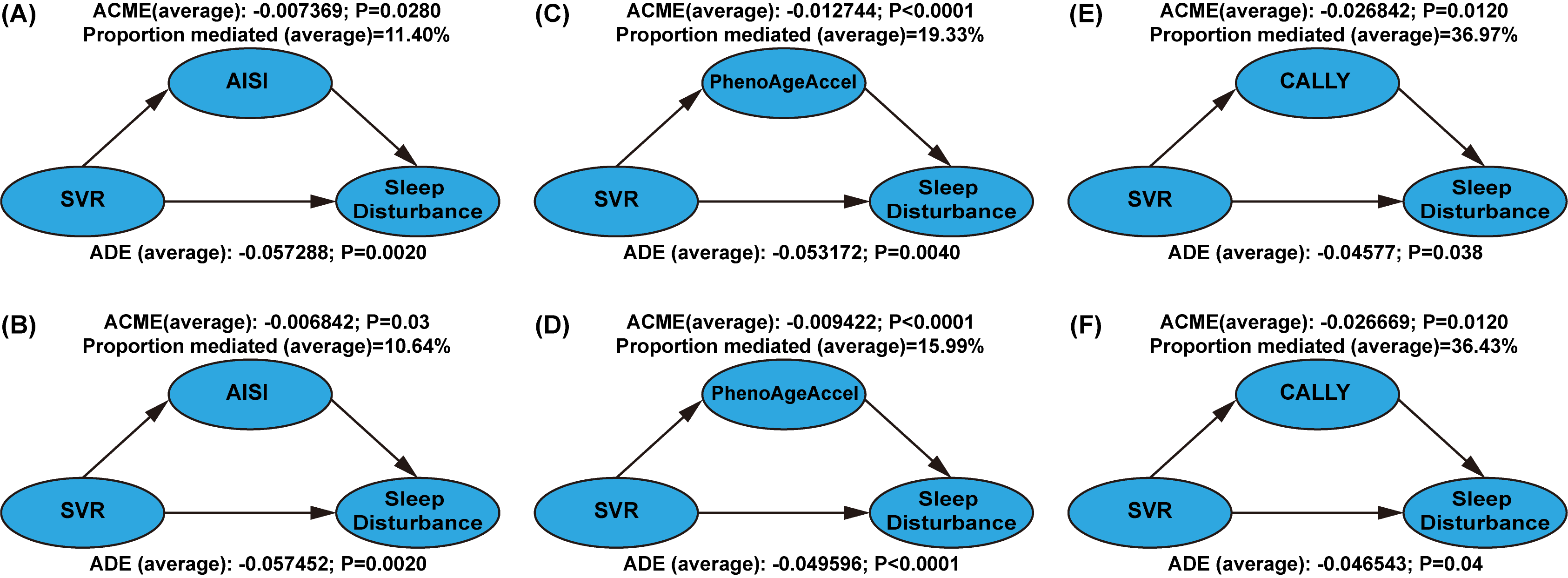

Supplement: Supplementary file 4 — Supplementary Material 4 [file 12888_2026_8248_MOESM4_ESM.tif]
